# Topotactic transformation of homogeneous phosphotungastomolybdic acid materials to heterogeneous solid acid catalyst for carbohydrate conversion to alkyl methylfurfural and alkyl levulinate†

**DOI:** 10.1039/c9ra03300a

**Published:** 2020-01-02

**Authors:** Dinesh Gupta, Chandrakant Mukesh, Kamal K. Pant

**Affiliations:** Chemical Engineering, Indian Institute of Technology Delhi New Delhi 110 016 India kkpant@chemical.iitd.ac.in dineshguptagkp@gmail.com

## Abstract

The strong interaction of higher transition metal oxides with inorganic non-metals can be promising for generating highly acidic three-dimensional materials by design. A comprehensive controlled acidity of heteropolyacid-like catalyst and interpretation of the microstructure and mechanism of the formation of a versatile heterogeneous solid acid catalyst, HPW_4_Mo_10_O_*x*_ has been heterogenized by biomass-derived cystine as organic linkers to control the acidity of as-synthesized materials, which have greater acidity and complexity in separation from the reaction mixture. The new and unique results obtained in catalysis done in biphasic reaction. Cystine binds to the surface of HPW_4_Mo_10_O_*x*_, and the topotactic transition occurred, change the morphology and lattice parameter. We described here a sustainable transformation of highly acidic (0.84 mmol g^−1^) heteropoly acid (HPW_4_Mo_10_O_*x*_) to cystine anchored on the active surface of the heteropoly acid and controlled the acidity (0.63 mmol g^−1^) and heterogenized the materials. As synthesized materials have been showing that for the direct formation of alkyl levulinate and furanics intermediate from carbohydrates. HPW_4_Mo_10_O_*x*_ and HPW_4_Mo_10_O_*x*_-Cys, act as acidic catalyst, and catalyse the mono- and disaccharides that are dissolved in primary and secondary alcohols to alkyl levulinate (AL) and alkyl methylfurfural at 170 °C under microwave irradiation with glucose as the substrate, AL yield reaches 62% with 84.95% selectivity. The catalyst can be easily recovered by filtration and minimum five times reused after calcination without any substantial change in the product selectivity. The analytical analysis of as-synthesis materials done by NH_3_-TPD, BET, XRD, FESEM, TEM, HRTEM, FTIR, ATR, TGA, DTA to stabilized the morphology and acidity controlled mechanism.

## Abstract 2

The strong interaction of higher transition metal oxides with inorganic non-metals can be promising for generating highly acidic three-dimensional materials by design.

## Graphical abstract

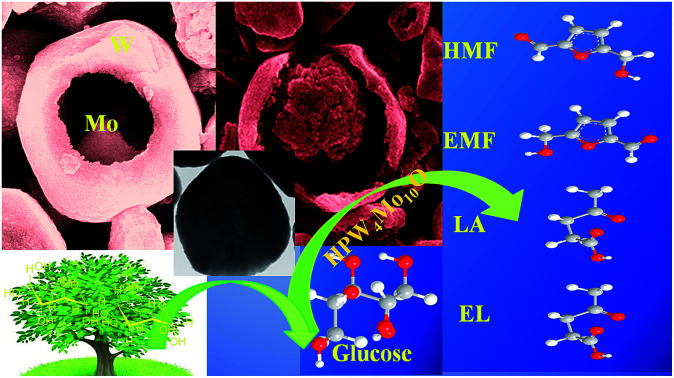

## 1. Introduction

Replacement of mineral acids, costly heterogeneous metal oxides and precious acidic ionic liquids by low-cost non-noble solid acid catalysts for the selective dehydration and rehydration of carbohydrates to alkyl levulinates and furanic intermediates holds tremendous promise for the clean and carbon neutral synthesis of biofuels and chemicals.^1^ Future unpredictable crude fossil fuels prices have stimulated interest in biomass-based fuels and chemicals synthesis as an alternative means to catalytically convert over functionalized carbohydrates (hexose and pentose sugars) into less oxygenated furanic fuels and chemicals.^2^ Glucose and fructose can be produced by the hydrolysis of cellulosic biomass by mineral acid or enzymatic pathways, which also renders the alkyl levulinate synthesis more sustainable.^3^ Commercial active catalysts usually involve mineral acids (HCl, H_2_SO_4_), dual acidic metal catalysts,^4,5^ metal salts^6^ and ionic liquids^7^ at a higher temperature. The thermal instability of carbohydrates is a major hurdle in this regard, and enzymatic hydrolysis,^8^ have been widely used because of their utility at low temperature. However, separation and cost of the enzyme is a major setback. On the other hand, specific and controlled materials synthesis, often improved process design, open the new route in higher product yield and selectivity at reduced costs of reaction. There are enormous opportunities for the design and synthesis of acidity controlled catalytic materials for the sustainable development of fuels and chemicals.^9^

Fossil-based petroleum products are currently major, gigantic players in chemicals industry for the synthesis of chemicals, fuels, and materials. However, pose foremost concerns in long smoothly future utilization due to day-by-day depletion of limited fossil resources and increasing energy demands, create huge pressure on fossil-based chemicals and fuels market.^10^ The search for alternative and sustainable energy is of crucial importance with the ever-growing population with increasing energy demands and environmental concerns, together with the limited reservoir of fossil fuels reserves.^11^ This is rise issued to the utilization of other sustainable and renewable substrate as an alternative to fossil fuels and chemicals. In this regards, lignocellulosic biomass, attract much more attention as an alternative to fossil fuels and chemicals. Direct utilization of lignocellulosic biomass has hurdles of its over-functionality and excess of oxygen. In this regards chemist and engineered designed environmentally sound reaction-design, cost-effective, and energy saving and atom efficiency chemical processes.^12^ Design of such type of process, catalyst always lined center-point to minimized the hazardous waste, increased the atom-efficiency and selectivity of product in an energy-efficient manner. Given these parameters, research endeavors directed towards utilization of metal salt as catalysts,^13,14^ but non-separation and hazardous impact on the environment is a major hurdle to effective utilization in the industry.

Recently heteropoly acid (HPA) materials attract more attention as solid acid catalysts possess and show strong Brønsted acidity.^15^ Studies at molecular level show that it have unique physicochemical properties, with their structural mobility and multi-functionality nature, these make it an ideal catalyst for the biofuels industry.^16^ Due to their higher acidity, and tunable acidic properties these are classified as a super acid^17^ because of its strong Brønsted acidity it showed vast application to acid catalysed reactions, HPA has attracted much interest. However, a key challenge that is often encountered is stabilization of HPA in the reaction medium and its higher activity. It is reported that the initial heat of ammonia adsorption was about 200 kJ mol^−1^ for H_3_PW_12_O_40,_ which is much higher than HZSM-5 and SiO_2_–Al_2_O_3_ catalyst.^17^ HPA functionalized with surface ligands can control the physical as well as chemical properties of the resulting materials and tuned the acidity, heterogeneity, composition, and size of the material. Moreover, the ligand interactions between active catalytic site can be used to control the acidity and interparticle distance and lattice symmetry.^18,19^ Another strategy is to anchor active metal center on a support with a stronger metal-support interaction. Previously, several many organmetallic^20^ as well as nanomaterials were loaded with HPA and used as an anchored homogeneous catalyst for selective hydrogenation.^21^ Many nano-particles are modified with HPA and used as an active catalyst for organic synthesis. TiO_2_ was modified with polyoxotungstates and used as photo-catalyst for dye degradation.^22^ Zhang *et al.* used phosphomolybdic acid for stabilizing and synthesis of a platinum single-atom catalyst and used as hydrogenation catalyst.^23^ Furthermore, by selecting and manipulating the surface properties of HPA, it would be possible to control the size and shape of the resulting heterogeneous materials. The potential for controlling the acidity by cystine a biomass fraction, in combination with the heterogenization of HPA materials, increases the “green” credentials of the catalytic reaction process, with higher selectivity, conversion, and yield of desired product. The catalyst recovery being proposed advantages over homogeneous highly active HPA catalyst. In this research work, we investigated the biomass-based cystine as effective linkers and binders for the controlling the acidity and preparation of heterogeneous HPA based materials for the selective conversion of alkyl levulinate and other furanics intermediate.

The effectiveness of group 6 metal chlorides, CrCl_3_-mediated microwave irradiation of cellulose and glucose, in IL as solvent explored by many previous articles and claimed to be energy-efficient and cost-effective for the conversion of biomass into platform chemicals and fuels additive.^24^ Brønsted acidic ionic liquids were used for HMF production from fructose.^25^ But, the hazardous nature of CrCl_3_ is a most significant issue to utilization as a catalyst. Chan *et al.* explored lower group-6 metal chlorides (WCl_6_) with anionic liquid in the biphasic solvent system at very low temperature (room temperature to 50 °C) to HMF synthesis.^26^ Recently molybdenum incorporated catalyst effectively explored for biomass conversion to value-added chemicals and fuels.^27,28^ However, many possible inorganic materials and a metal oxide that consist of three or more elements are still mislaid from previously available research articles. A few of these materials composition are mislaid because they have not been prepared yet, whereas others are mislaid because of the inconsistent into competing phases.^29^ Oluwafemi *et al.*^30^, used l-cysteine ethyl ester hydrochloride as a capping agent for the synthesis of colloidal CdSe nanoparticles. Mntungwa^31^*et al.* investigated the effect of capping agent (l-cysteine ethyl ester hydrochloride) on the size, structure, and morphology of the as-synthesized nanoparticles. Rahme^32^*et al.* reported that synthesis of AuNPs in the presence of l-cysteine methyl ester hydrochloride as a capping agent and stored materials for 12 months no significant changes observed and reported high stability of the colloidal solution. These above findings indicate that the interesting properties of cysteine can be successfully applied toward capping agent for heterogenization of materials. The several hetrogeneuous and homogeneous catalyst were reported for the preparation of AL, AMF and their derivatives from cellulose or biomass derived building block molecules.^33–35^ Magnetically recyclable carbonaceous solid acid was used for syntheis of 5-ethoxymethylfurfural (EMF) from carbohydrates.^36^ Zhang *et al.* were used aluminum-based mixed-acid to intensify the yield of EMF.^37^ ALs fined application in different sectors of the chemical industry, like biofuels additives, bio-solvents, an additive for odorous, plasticizing agent and as building blocks for various chemical transformations.^4,6,9,38^ AMF not only decreases the boiling point but also preserving an increased the calorific value.^39^ Levulinic acid (LA), methyl levulinate (ML), ethyl levulinate (EL), propyl levulinate (PL) and butyl levulinate (BL) have a potentially versatile building block for the synthesis of several other chemicals and excellent fuel additives properties.^40^ As reported, EMF is an excellent fuel additive for diesel with high energy density (8.7 kW h L^−1^), similar to gasoline (8.8 kW h L^−1^), diesel (9.7 kW h L^−1^) and higher than ethanol (6.1 kW h L^−1^).^41^ Lew *et al.* reported similar energy density of EMF 30.3 MJ L^−1^ (8.42 kW h L^−1^) and other fuels like gasoline 31.1 MJ L^−1^ (8.64 kW h L^−1^), diesel 33.6 MJ L^−1^ (9.34 kW h L^−1^) and ethanol 23.5 MJ L^−1^ (6.53).^42^ In the NREL technical report mentioned the energy density of EL 24.8 MJ kg^−1^ and butyl levulinate is 27.1 MJ kg^−1^, which is show vast potential of this biomass-derived oxygenates to use as alternative fuels.^43^ Currently, there were several methods of the preparation of EMF and EL reported. It is undoubted that the etherification of the hydroxyl group in HMF or furfuryl alcohol with ethanol is the effective route. However, the economical, selective and practically large-scale process of EMF or AMF from HMF is limited due to the high production and separation cost of HMF. The motivation of this work is to explore the composition of mislaid materials composition and controlling the acidity of prepared materials by using a renewable binder. This preparation of materials in the field of catalysis-synthetic solid acid chemistry is also important because of the vast potential to controlled the selectivity of desired products and easily separately by reaction mixture.

Understanding the basic properties of heteropoly acids and capping of ligands will help in developing new types of solid acid materials with improved properties which should lead to, for example, dehydration, etherification and hydrogenolysis more effectively, more selectively and more efficiently. These should be capable of efficient dehydration and etherification of carbohydrate to biofuels which could help us in our future design of biobased fuels and chemicals at moderate cost and environmentally friendly manner.

## 2. Experimental section

### 2.1 Materials

All chemicals were of analytical purity and used without purification. Sucrose, fructose, glucose, xylose, were purchased from Fisher Scientific Chemicals (India) and used as received. DMSO-d_6_, CDCl_3_, 5-hydroxymethylfurfural, furfural, furfuryl alcohol, levulinic acid, ethyl levulinate are purchased from Sigma-Aldrich chemicals, India with high purity and used as received. Methyl isobutyl ketone (MIBK), tetrahydrofuran (THF), ethanol, methanol, 2-propanol, *n*-butanol are purchased from, Merck chemical India, K_2_HPO_4_ from Qualigens, Na_2_WO_4_·2H_2_O from a Central Drug House, and Na_2_MoO_4_·2H_2_O from Merck, India, purchased.

### 2.2 Material preparation

Two types of heteropoly acidic materials prepared (i), HPW_4_Mo_10_O_*x*_ and (ii) HPW_4_Mo_10_O_*x*_-Cys was prepared in a two-step. In first step 1 equivalent dehydrated K_2_HPO_4_ dissolved in 50 mL hot water, labeled solution (A). In another 100 mL glass jacket, 4 equivalent of Na_2_WO_4_·2H_2_O dissolved in 50 mL hot water and labeled solution (B). Solution (B), dropwise added in solution (A) and stirred for 5 min at the hot condition, then cool the reaction mixture in an ice bath, then 2.5 mL concentrated H_2_SO_4_ added as precipitating agent. After addition of H_2_SO_4,_ white precipitate form. In second step 10 equivalent of Na_2_MoO_4_·2H_2_O dissolved in 60 mL distil water, stirred at room temperature for 5 min, then dropwise added in the first step solution, after addition of ∼5 mL, white colour disappeared, then continuous addition, colourless solution obtained, the final solution composition is 1 : 4 : 10 (K_2_HPO_4_ : Na_2_WO_4_·2H_2_O : Na_2_MoO_4_·2H_2_O). In colorless solution, one drop chilled H_2_SO_4_ added green precipitate formed, after shaking green precipitate dissolved, then continuous dropwise added till the green-yellowish precipitate persist. After that nitrogen gas was passed at the bottom for 1 h, then filtered and washed with water followed by washing with methanol and drying under vacuum in the presence of nitrogen at 120 °C. As prepared materials is denoted as HPW_4_Mo_10_O_*x*._

(ii) Preparation of HPW_4_Mo_10_O_*x*_-Cys: for the synthesis of HPW_4_Mo_10_O_*x*_, with a controlled acidity and heterogenization, l-cysteine ethyl ester hydrochloride was used as a binder. In a typical synthesis of HPW_4_Mo_10_O_*x*_-Cys, step 1 and step 2 are same as HPW_4_Mo_10_O_*x*_ only here ammonium molybdate used as a precursor in place of sodium molybdate. In step 2, 1.5 equivalent of l-cysteine ethyl ester hydrochloride dissolved in 20 mL (olyal alcohol and oleic acid 1 : 1 v/v) added drop-wise and reaction mixture stirred at room temperature to 240 °C, colour change yellow then 2 mL distil water added, reddish-brown precipitate obtained, 0.5 mL chilled H_2_SO_4_ added as precipitating agent, and more precipitate obtained, separated by acetone, filter and wash with water and methanol, dry at 250 °C and labelled as HPW_4_Mo_10_O_*x*_-Cys.

### 2.3 Characterization

Powder XRD patterns of the as-synthesized materials were collected room temperature with a Phillips X Pert diffractometer PW 1390 Cu-Kα radiation (*λ* = 1.54056 Å) source operating at 40 kV and 30 mA. The dry powder was spread on the top of a glass substrate and was then measured in the reflection geometry. Transmission Electron Microscopes (TEMs) and high-resolution transmission electron microscopes (HRTEM) were performed with a JEOL 120 kV equipped with LaB6/tungsten filaments and the FEI Tecnai TF20, at accelerating voltage of 200 kV respectively. Samples suitable for TEM observation were prepared by applying one tiny drop of sonicated materials onto the carbon coated Cu grid and allowing the solvent to slowly evaporate at room temperature. Scanning electron micrographs (SEMs) for as synthesis materials were obtained with a Zeiss EVO 50 & EVO 18 and Field Emission Scanning Electron Microscope (FESEM) with an FEI Quanta 200. Nitrogen adsorption isotherms were obtained with a Micromeritics ASAP 2010 at 77 K. Fourier transform infrared spectroscopy (FTIR) spectra of as-synthesized materials were recorded with a Fisher Scientific Nicolet iS50 spectrophotometer (2 cm^−1^ resolution and 32 scans) in dried KBr pellets and a measuring range of 400–4000 cm^−1^. For the total acidity calculation of as-synthesis materials were measured by NH_3_-TPD analysis was performed on Micromeritics, ChemiSorb 2720 set-up equipped with a thermal conductivity detector. Surface area of sample was determined by BET principle and pore parameters of the samples were determined by BJH method with the help of Micrometrics ASAP 2010 at 77 K. Raman analysis was done with a Renishaw inVia Raman Microscope spectrometer equipped with a laser beam emitting at 514 and 785 nm. ^1^H and ^13^C NMR done by Bruker Avance AV-III with 400 MHz spectrometers.

### 2.4 Performance of as-synthesis catalyst in a microwave reactor

The dehydration and etherification reactions of mono-sugars were carried out by charging substrates, solvent, and catalyst in a 10 mL and 100 mL Teflon tube fitted in the ceramic jacket, microwave tubes. The loaded microwave tube was closed then inserted into the programmable multiwave pro microwave reactor and set the desired reaction temperature and time. After completion of the reaction at the desired time opened and reaction mixture firstly filtered with whatman filter paper and separated the catalyst. For GC and HPLC analysis, solutions were filtered through a 0.22 μm cut of syringe filter. The quantification of the reaction product, reaction mixture analyzed by GC and HPLC in the presence of internal standard and purity of product confirmed by ^1^HNMR, using mesitylene as an internal standard.

## 3. Results and discussion

### 3.1 Ligand exchange-induced phase transformation of homogeneous HPW_4_Mo_10_O_*x*_ into heterogeneous HPW_4_Mo_10_O_*x*_-Cys

To reveal the nature of active sites that favor the selective dehydration-rehydration and etherified products, multiple characterizations techniques were used to investigate the functionality and structure of the multifunctional catalyst. After using the capping agent cystine, change the number of active acidic sites of as-synthesized materials, which enhances the selectivity of the desired product. Our approach to heterogenized and controlling the acidity of HPA affords various advantages compared to conventional heterogeneous catalysis: (i) the catalyst system can show greater reactivity and selectivity like a homogeneous catalyst; (ii) unlike most homogeneous catalysts with comparable performance, the heterogenized HPW_4_Mo_10_O_*x*_-Cys can be easily recycled; (iii) significantly enhanced the selectivity of the desired product and stopped the rehydration of furanics. Herein, we provided future direction for the heterogenization of HPA and it used for the efficient solid acid catalyst. Cysteine has active amine functional group to be bound to the acidic sites and control the acidity and also heterogenization the materials. The deposition and anchoring of cystine on the active sites of HPW_4_Mo_10_O_*x*_ resulted from the ionic interaction between the amine groups of cysteine with a metalcenter. This interaction prevented the leaching of metal in the reaction mixture even under hot-liquid phase reaction condition under microwave irradiation. The supported material displayed no detectable leaching of metals under hot-plate filtration test and in this regards functioned as true heterogeneous catalysts. However, the tunable acidity of the as-synthesized materials rendered these materials effective in a variety of other transformation. Moreover, there is an increasing interest in the utilization of solid acid catalyst in place of mineral acid, metal particles, both as core–shell structures or embedded in matrices, since the HPW_4_Mo_10_O_*x*_-Cys gives an extra degree of freedom to control and tailor the acidic properties.

**Fig. 1:**
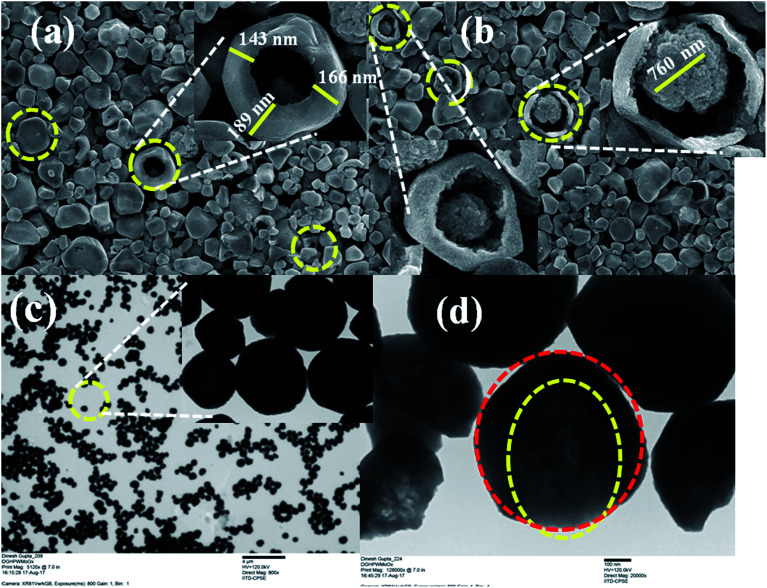
Representative SEM and TEM images of monodispersed hollow core–shell particles of as-synthesized HPW_4_Mo_10_O_*x*_ materials: (a) and (b) SEM images, scale bar 1 μm; (c) and (d) TEM images, scale bar 4 μm and 100 nm respectively.

**Fig. 2:**
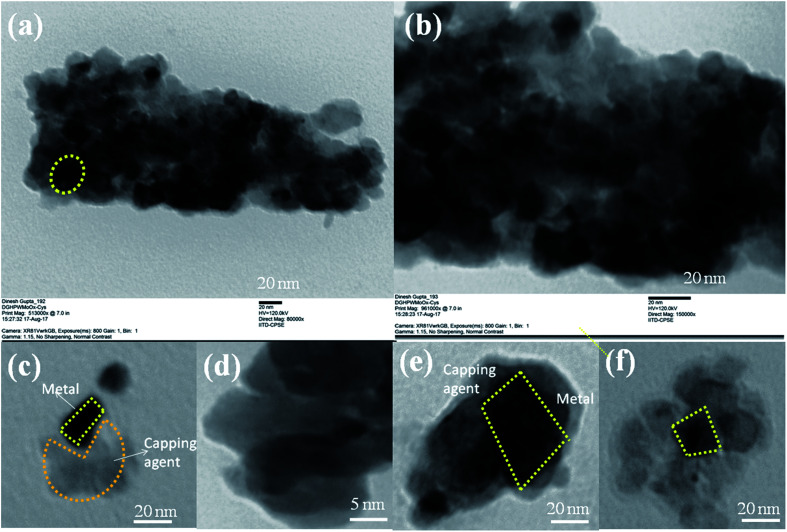
Structural and morphological characterization of the as-synthesis HPW_4_Mo_10_O_*x*_-Cys by TEM.

The l-cysteine ethyl ester hydrochloride was capped on the HPW_4_Mo_10_O_*x*_ surface after the synthesis ofHPW_4_Mo_10_O_*x*_. As synthesis, catalyst showed 140–170 nm outer cores with 600–700 nm total materials size. As-synthesis material was composed of nano-sized MoO_*x*_, WO_*x*_, and incorporated with phosphorous and hydrogen determined by various techniques before and after the capping the cysteine at the surface transformation. Characterization of as-synthesized materials from TEM and HRTEM are shown in Fig. 1, offered direct visualization of the morphology of the as synthesis materials, which looks like core–shell type of morphology adopted by materials. The HPW_4_Mo_10_O_*x*_ possessed reproducibly uniform size and shape materials; the low-magnification TEM (Fig. 1c, 4 μm) image indicates excellent particle uniformity. The electron contrast between the cores and shells in the high-resolution (Fig. 1d, 100 nm) and insect of Fig. 1a and b, confirms the formation of hollow particles. The SEM image is shown in Fig. 1a and 1b (inset) demonstrates that the as-synthesis materials adopted hollow-core–shell morphology and could be prepared. Both the SEM image at a higher magnification (Fig. 1a and b) and TEM image (Fig. 1d) reveal that the hollow-core–shell were uniform in diameter and closed pack in arrangement during the sample preparation. To examine the morphology of as-synthesized materials, without capping agent and after the capping, results show in Fig. 1 and 2. Interestingly particles of two different morphologies with different sizes have been observed. Large hexagonal hollow (3D) with average diameter 0.6–0.7 μm (edge to edge) and thickness of wall ∼150 nm are seen in Fig. 1a. However, the as-synthesized HPW_4_Mo_10_O_*x*_-Cys is small in diameter and quasi-rhombus in morphology (Fig. 2). The average particle diameter of these nanoparticles is 25 nm. cystine as capping agent favors the formation of thermodynamically more stable shape, stability in nature.

When l-cysteine ethyl ester hydrochloride used as capping agent with oleyl alcohol and oleic acid, ∼28 times size of metal particles reduce as shown in Fig. 2. Similar nanoparticle size was observed as previous report.^31^Fig. 2 shows TEM images of as-synthesized HPW_4_Mo_10_O_*x*_-Cys particles capped with cysteine. Fig. 2c–e show that cysteine capped HPW_4_Mo_10_O_*x*_ particles are quasi-rhombus, with an average size 25 nm. The corresponding some high-resolution TEM image (Fig. 2c and d) of the cysteine capped HPW_4_Mo_10_O_*x*_ nanoparticles, reveals the lock-key like an adaptation of materials. TEM images of the HPW_4_Mo_10_O_*x*_-Cys (Fig. 2) easily visible that the metal particles embedded by the capping agent. Electron prove-micro analysis (EPMA) and energy dispersive X-ray analysis (EDX) enabled quantification of the metal content in the as-synthesized materials and the extent of metal leaching during the reactions. X-ray photoelectron spectroscopy was utilized to determine the nature of metal oxidation state within the materials.

**Fig. 3:**
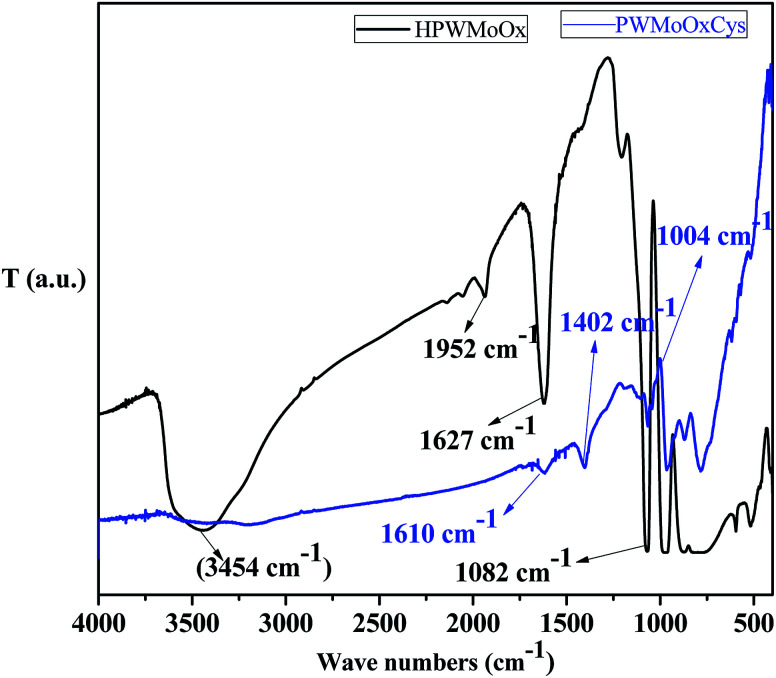
FT-IR spectra of as-synthesized HPW_4_Mo_10_O_*x*_, and HPW_4_Mo_10_O_*x*_-Cys materials.

The FT-IR spectra of as-synthesis HPW_4_Mo_10_O_*x*_ and HPW_4_Mo_10_O_*x*_-Cys materials were to stabilize the acid strength changes due to the free and binding state of inorganic oxygen-containing anion^44^ are shown in Fig. 3. As it is well-stabilized the fact that heteropolyanion would be strongly adsorbed into supports.^44^ In the HPW_4_Mo_10_O_*x*_, the broad peak has been investigated may be due to a hydroxyl group (3600–3400 cm^−1^)^45^ was assigned, which is missing in the HPW_4_Mo_10_O_*x*_-Cys spectrum, indicate that hydroxyl group protected or bind with cysteine moiety. The characteristics peaks of HPA shown at 1082 cm^−1^ may be due to P–O interaction, metal oxygen bridged at 800–900 cm^−1^ range may be due to W–O–W^46^ and Mo–O–Mo asymmetric vibration, associated to the typical Keggin anions. At three decade ago, Nomiya *et al.*^47^, reported that immobilized HPA in the 1100–700 cm^−1^ showed four stretching vibration frequency due to M-terminal oxygen, M–O–M octahedral edge, corner-sharing's and heteroatom (X)-triply bridged oxygen. Khder *et al.*,^48^ reported that band at 1081, 982, 889, 797 and 595 cm^−1^ assigned to the stretching vibrations of P–O, W–O_t_, W–O_c_–C, W–O_e_–W and bending vibration of P–O, respectively. Popa^49^*et al.* reported that stretching vibration at 965 cm^−1^ came due to Mo–O_c_–Mo binding and 595 cm^−1^ arise for the bending vibration P–O. In literature, well stabilized that the bands in the range of 400–1000 cm^−1^ are attributed to the stretching and bending vibrations of metal–oxygen characteristics.^49^ The absorptions band located at 711, 600 and 523 cm^−1^ are attributed to the Mo–O stretching vibration, reported by Song *et al.*^50^ They also reported that band at ∼1000 cm^−1^ is related to the terminal oxygen atoms of Mo. We are also observed the approximately same result in HPW_4_Mo_10_O_*x*_ materials, but after capping of cystine minimized the characteristics of HPA peaks. Additionally, peak at 1610 and 1627 cm^−1^ may be due to C=O starching. Similarly as for HPW_4_Mo_10_O_*x*_-Cys materials, resulting in the lowering the characteristic FTIR vibrations, and another 896 and 831 cm^−1^ vibration spectra were observed.

**Fig. 4:**
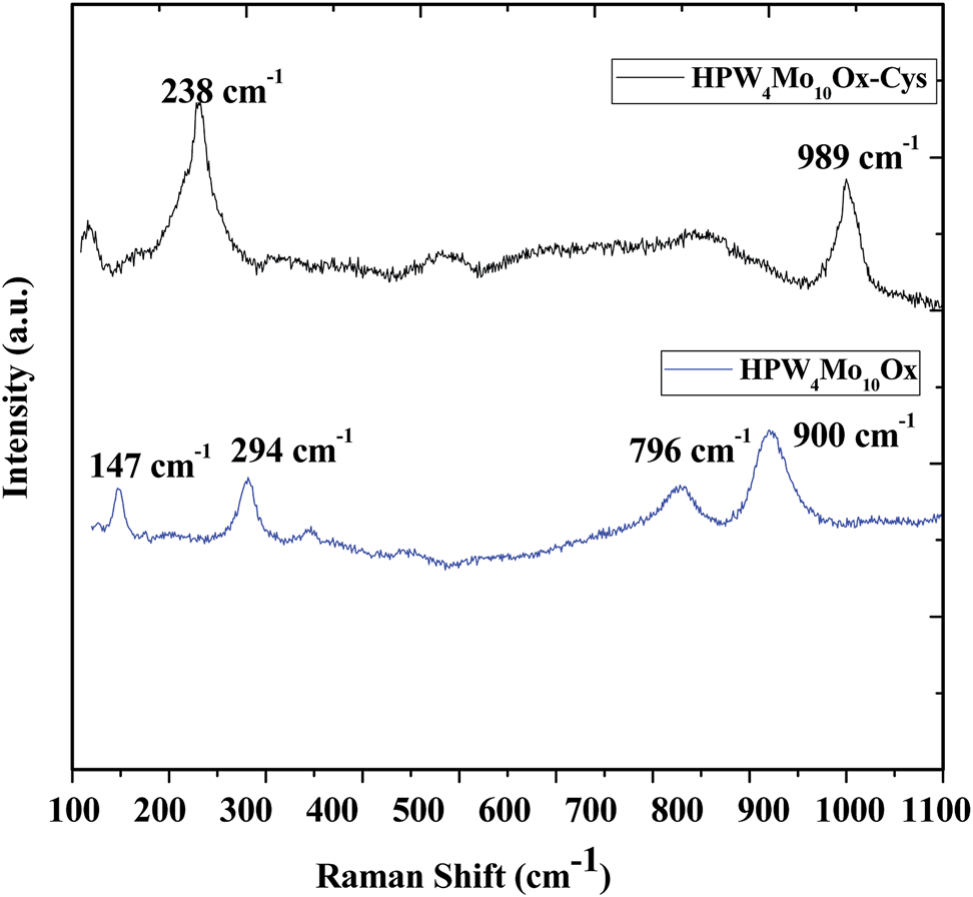
Raman spectra of as-synthesized HPW_4_Mo_10_O_*x*_, and HPW_4_Mo_10_O_*x*_-Cys materials.

Raman scattering spectroscopy was useful to study the original Keggin structure and deformation in structure due to support, because it is extremely sensitive to the Keggin unit, and the support has no significant interference on the Raman scattering originated from the Keggin structure. Guo *et al.*^51^, reported that P–O bonds correspond to stretching vibrations at 1009.5 cm^−1^, W=O bonds (993.9 cm^−1^) and W–O–W bonds correspond to 912.4 cm^−1^. The investigation also revealed that shifts of the peak position are possible due to the strong interaction between the Keggin unit and the support. As Fig. 4, show that peak at 989 cm^−1^ and 900 cm^−1^ may correspond to W=O bonds W–O–W linkage but peak at near 1010 cm^−1^ peaks is missing, which indicates that as-synthesized materials don't contain Keggin unit. Devassy *et al.*^52^, reported that band at 998 and 974 cm^−1^could be attributed to W=O symmetric and asymmetric stretching modes. They also claim that broad peak at 893 cm^−1^ can be assigned due to W–O–W asymmetric stretching mode. Popa *et al.*^49^, reported that symmetric and asymmetric vibrations of terminal oxygen (Mo–O_t_) and corner shared bridged oxygen Mo–O–Mo are lies at 983, 882, 246 and 154 cm^−1^. In the literature reported that peaks at the lower range might be due to Mo–O interaction. In current work, Mo also interferes the lattice. Therefore two peaks at 796 cm^−1^ and 900 cm^−1^ observed, which is shifted than the characteristics peak of WO_3_ (802 and 716 cm^−1^),^53^ indicate that different bonding mode of W.

**Fig. 5:**
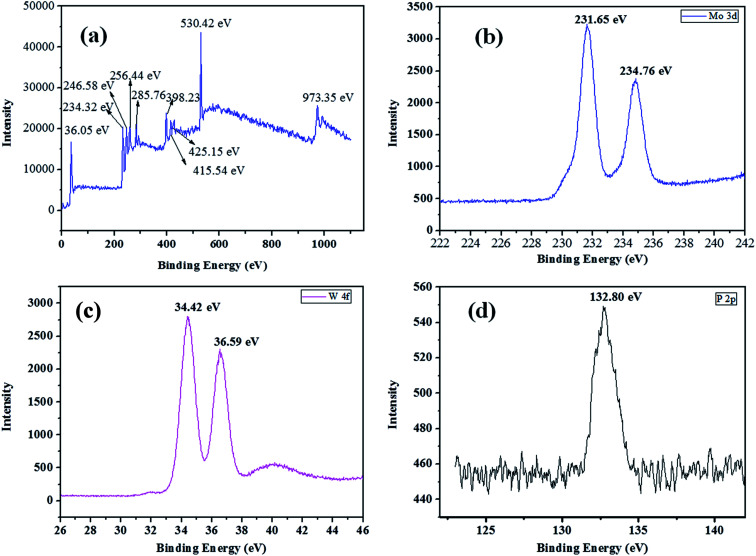
XPS survey spectrum (a), 3d core-level spectrum of molybdenum (b), C 1s core level spectrum of carbon in the synthesized material (c), P 2p core level spectrum of phosphorous (d).

The X-ray photoelectron spectroscopy was used to determine the chemical bonding, the valence state of W, Mo and surface composition of as-synthesized HPW_4_Mo_10_O_*x*_ materials. The XPS survey spectrum of materials was shown in the Fig. 5a. The survey spectrum shows peaks for W, Mo, C, O, S and P. The Mo 3d core level spectrum show 3d_3/2_ and Mo 3d_5/2_ binding energy for as-synthesis materials is observed at 231.65 eV and 234.76 eV with spin–orbit separation of 3.11 eV and Mo d_3/2_/Mo_5/2_ ratio of 0.73, as shown in Fig. 5b, which is lesser than the typical characteristics peak of Mo^6+^and indicate the reduced form of Mo.^54^ The XPS high-resolution spectra of W 4f core levels for the as-synthesized materials shown in Fig. 5c. The binding energy for W 4f_7/2_ and W 4f_5/2_ levels are observed at 34.42 eV and 36.59 eV respectively, with spin–orbit separation of 2.17 eV and the W 4f_5/2_/W 4f_7/2_ ratio of 0.814. The energy position of the doublet is lesser than the W^6+^ reported in the literature^55^ and best matched with W^5+^, and indicted that W is attached with hydrogen.^55^ The XPS high-resolution spectra of P 2p core levels show the binding energy 132.80 eV in Fig. 5d, indicated that P–O linkage in the as-synthesized materials.^55^ Combined with FTIR, XRD, and TEM analysis, it is clear that the Mo ions as the doping were successfully incorporated into the crystal lattice of WO_3_ and maybe W–Mo boding formed, because bonding energy of W and Mo had a trend to lower, suggesting the presence of W^5+^ and Mo^5+^.^56^ Shpak *et al.*, reported binding energy for W 4f_7/2_ and W 4f_5/2_ levels of tungsten atoms for W^5+^-sates of oxide (34.8 eV), well matched with our as-synthesized catalyst.^57^ The narrow scan of as-synthesis materials shows the O 1s core level located at 530.42 eV, which was different than the pure WO_3_ (529.4 eV)^56^ trends to different nature of W–O bonding. Shpak *et al.*, also reported that binding energy of O 1s (530.6 eV) correspond to O1-levels of oxygen atoms O^2−^ in the synthesized materials^57^ and may be the composition written as W_*x*_^5+^W_1−*x*_^6+^O_3−*x*._Li *et al.*^58^, reported that peaks at 231.7 eV and 234 eV belongs to Mo^5+^ oxidation state, which is close to (231.65 and 234.65 eV) our as-synthesis material.

**Fig. 6:**
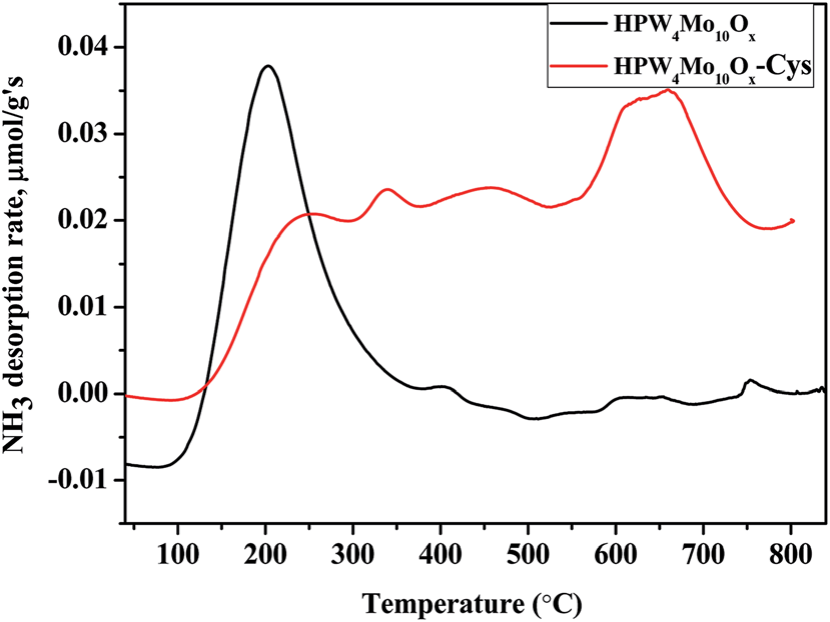
NH_3_-TPD profiles for the as-synthesized HPW_4_Mo_10_O_*x*_, and HPW_4_Mo_10_O_*x*_-Cys catalyst.

Acidity analysis of as-synthesis HPW_4_Mo_10_O_*x*_, and HPW_4_Mo_10_O_*x*_-Cys material at 750 °C, shows much higher total acidity (0.84 mmol g^−1^ and 0.63 mmol g^−1^, respectively) from temperature programmed desorption of ammonia (TPDA). Which is much higher than the alumina-supported catalyst (0.34 mmol g^−1^) and silica (0.07 mmol g^−1^).^59^ Alhanash *et al.*,^60^ reported acidity strength on the basis of TPDA peaks at different temperature range. They mentioned that absorption peaks below 300 °C correspond to weak acidity, above 300 °C, moderate acidity and the peaks at more than 500 °C due to the stronger acidity of materials. As-synthesis materials (HPW_4_Mo_10_O_*x*_), show a strong peak at lower acidity range (Fig. 6) and HPW_4_Mo_10_O_*x*_-Cys show a strong peak at higher temperature range. On the basis of calibrations data, total adsorption of NH_3_ for every peak find-out by instrument and acidity for each peak calculated. HPW_4_Mo_10_O_*x*_, show three peaks at 203, 394 and 753 °C and contributed 0.79, 0.046 and 0.0075 mmol g^−1^ respectively. HPW_4_Mo_10_O_*x*_-Cys show four peaks at 340, 224, 454, 659 °C and contributed 0.15, 0.037, 0.15 and 0.29 mmol g^−1^ respectively. As graph (Fig. 6) and calculation of acidity trend indicate that after using the capping agent, decreased (0.84 to 0.63 mmol g^−1^) the total acidity and most significantly, change the trend of acidity pattern. Without the capping agent as-synthesized poly-metallic acidic catalyst show strong peaks and acidity strength (0.79 mmol g^−1^) at a lower temperature (203 °C) range, while after the capping, strong strength (0.29 mmol g^−1^) show at 659 °C. The change of material acidity nature provides to design the more selective synthesis of a desired product.

A wide range of etherification, dehydration and rehydration reactions can be efficiently catalyzed by these materials, which can be designed to the synthesis of control acidity as well as high degrees of reaction selectivity of the desired product. The elemental analysis of as synthesis materials was performed with the help of electron probe micro-analyser (EPMA) with LaB6 gun as shown in Tables S1 and S2.† Tables S1 and S2† show the 10-point analysis of as-synthesis materials with ranges minimum-to maximum 0.49% to 1.79 mol% of phosphorous, 4.04 to 22.33 mol% of oxygen, 0 to 1.49 mol% of nitrogen, 8.84 to 35.6 mol% of molybdenum, 6.66 to 28.02 mol% of tungsten in HPW_4_Mo_10_O_*x*_-Cys catalyst. As-synthesis HPW_4_Mo_10_O_*x*_ catalyst show minimum-to maximum 1.13% to 1.47 mol% of phosphorous, 10.76 to 25.68 mol% of oxygen, 22.7 to 29.7 mol% of molybdenum, 28.02 to 37.20 mol% of tungsten. The estimated value of the metal higher and in regular pattern in HPW_4_Mo_10_O_*x*_ compare to HPW_4_Mo_10_O_*x*_-Cys.

**Fig. 7:**
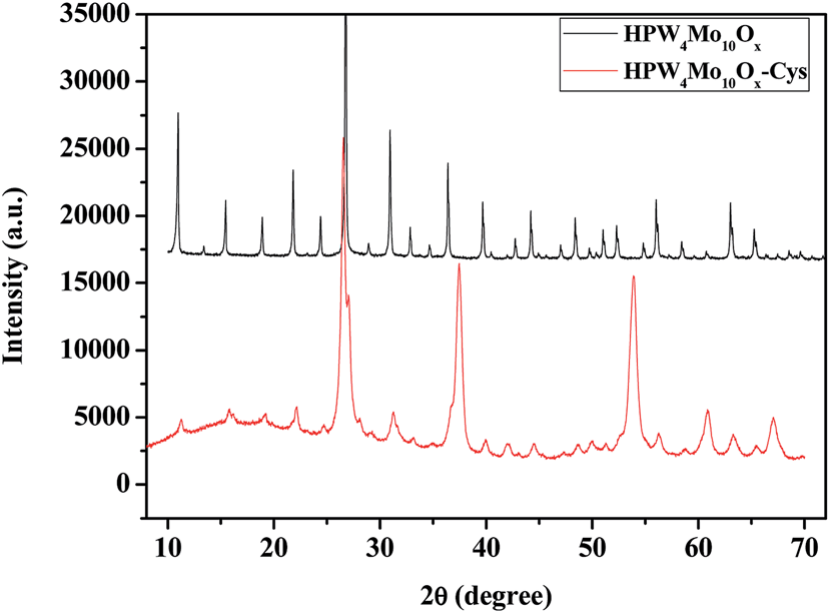
Powder-XRD patterns of as-synthesized samples.

Fig. 7 shows the powder-X-ray diffraction patterns of as-synthesized materials with the capping agent and without the capping agent. As far as the as-synthesized materials with capping agent (HPW_4_Mo_10_O_*x*_-Cys), show broad characteristics peaks of metal oxide indicate that nano-range particles are formed. Without capping agent materials (HPW_4_Mo_10_O_*x*_) show that various intensive and sharp peaks at corresponding 2*θ* value and respective *d*-spacing of the lattice fringes was found to be, 10.91 (0.81 nm), 15.0 (0.59 nm), 18.88 (0.47 nm), 21.85 (0.41 nm), 24.35 (0.36 nm), 26.79 (0.33 nm), 30.93 (0.29 nm), 32.79 (0.27 nm), 36.35 (0.25 nm), 39.58 (0.22 nm), 42.77 (0.21 nm), 44.20 (0.20 nm), 47.02 (0.193 nm), 48.45 (0.19 nm), 51.05 (1.79 nm), 52.33 (0.175 nm), 54.88 (0.17 nm), 56.10 (0.16 nm), 58.49 (0.16 nm), 63.11 (0.15 nm), and 65.39 (0.14 nm). In literature peak at 23.12 (002), 23.58 (020) and 24.38 (200) correspond to WO_3_ and 20.48 (111), 23.58 (020) for the WO_3_·H_2_O. As XRD pattern of HPW_4_Mo_10_O_*x*_-Cys indicate that the broadening of XRD peaks distinctly reflects the nanocrystalline nature of the materials which is more pronounced in the case of HPW_4_Mo_10_O_*x*_-Cys materials. In our synthesis method, an excess of cystine, oleic acid and oley alcohol was used to ensure stabilization and restricting the size of the particles in the nano range. The XRD pattern of as-synthesis HPW_4_Mo_10_O_*x*_-Cys show reflections at 2*θ* with respective *d*-spacing of the lattice fringes was found to be at 22.14 (0.40 nm), 26.45 (0.34 nm), 31.89 (0.28 nm), 37.45 (0.24 nm), 53.97 (0.17 nm), 60.93 (0.15 nm), 63.29 (0.15 nm), 67.14 (0.14 nm).

To further calculate the crystallite sizes of as-synthesized materials by Scherrer equation, which indicate that peak width is inversely proportional to crystallite size. In heterogenized materials HPW_4_Mo_10_O_*x*_-Cys, show broad reflection peaks and indicate the nano-range. As per calculation its show 10 to 30 nm size of metal nanoparticles. As an obtained result was found to be in good agreement with the size evaluated from TEM and prove that topotactic conversion can be achieved by capping agent and also reducing the particle size. The generation of nano-range particles through the process of topotactic intercalation changes the arrangement of particles that can enhance the activity and selectivity of furanics products. We believe that nano-range particles assembly and controlling the acidity are these two advances promise to synthesis and modification of heteropoly anion with tunable physical properties create new facet in catalysis.

**Fig. 8:**
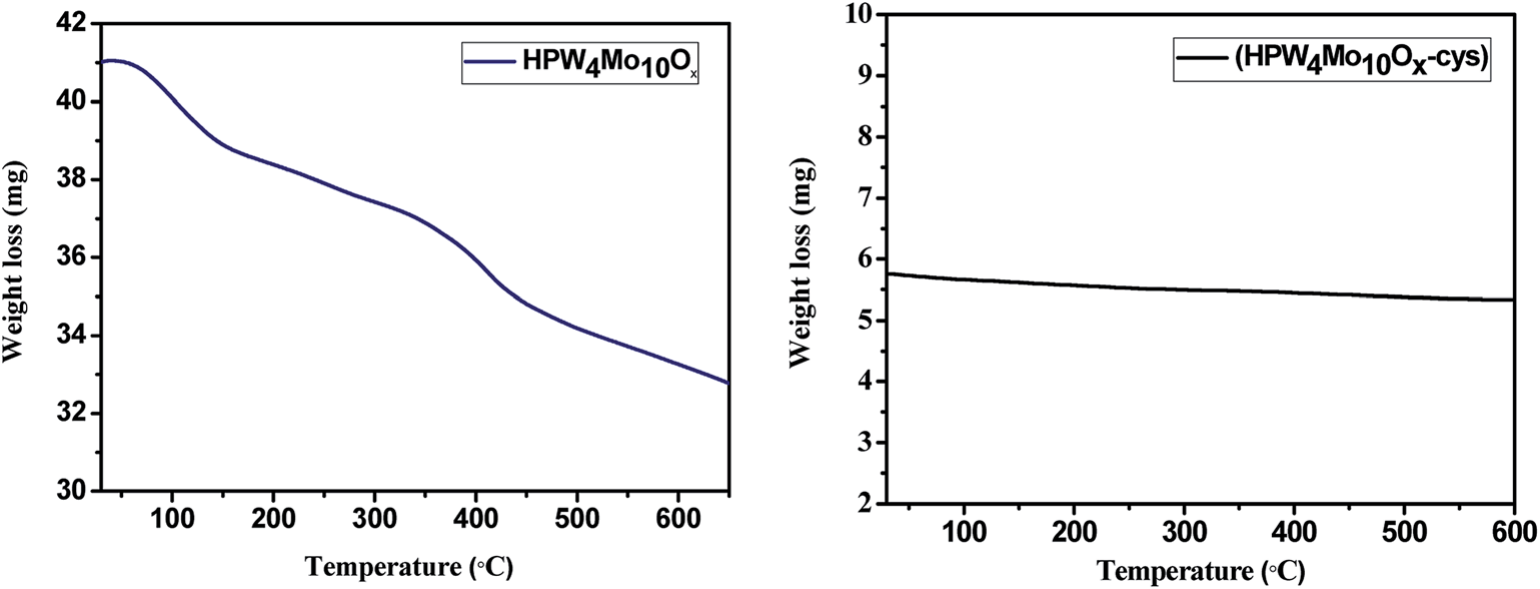
TG curves of as-synthesized materials of HPW_4_Mo_10_O_*x*_ and HPW_4_Mo_10_O_*x*_-Cys.

Fig. 8 depicts the thermogravimetric curves of the HPW_4_Mo_10_O_*x*_ and HPW_4_Mo_10_O_*x*_-Cys materials. The HPW_4_Mo_10_O_*x*_ show 24.39 weight%, mass loss whereas HPW_4_Mo_10_O_*x*_-Cys only 5.2% weight loss from room temperature to 600 °C, result indicate that HPW_4_Mo_10_O_*x*_-Cys, show higher thermal stability in compared to without capping agent. At initial weight loss were attributed to the loss of physically-adsorbed and chemically bonded water.

### 3.2 Catalytic dehydration and rehydration of mono-sugars

**Scheme 1:**
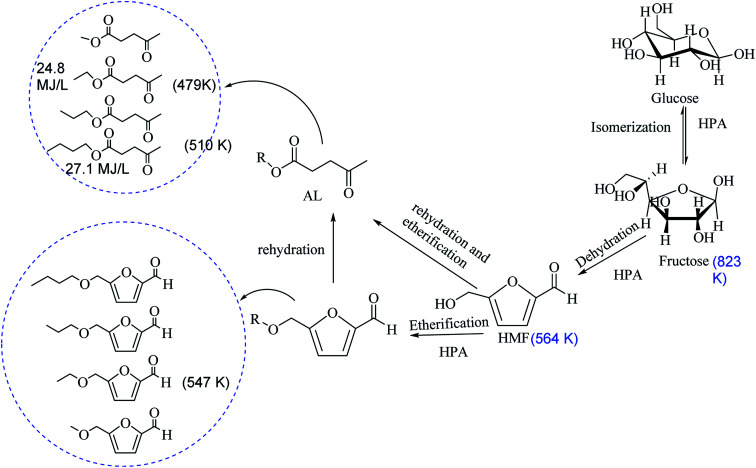
Decreasing of boiling-point after removable of an oxygen atom from fructose.

The basis for dehydration, etherification and rehydration of carbohydrate to 5-hydroxymethylfurfural (HMF), 5-alkoxymethylfurfural (AMF) and alkyl levulinates (ALs) are outlined in Scheme 1. We explored economically feasible mono-sugar (glucose) for the one-pot dehydration and etherification of respective AMF and AL. Dehydration and etherification of glucose into HMF and the etherification of HMF to AL are acid-catalyzed reactions, it rationally proceeds into two separable reactions into the one-pot reaction.

### 3.3 Dehydration and etherification of glucose

We initially prepared HPW_4_Mo_10_O_*x*_ and HPW_4_Mo_10_O_*x*_-Cys macro and nanocatalysts respectively by a simple one-pot synthesis process and then applied it to glucose dehydration and etherification of reaction under microwave irradiation. As shown in Table 1 (entry 1), HPW_4_Mo_10_O_*x*_ catalyst exhibited 22.45% selectivity to HMF, 42.68% selectivity to EMF, 12.82% selectivity to EL as well as a formylated and other products 6.10% at a glucose conversion of 84% and isolated mol% yield (56%). Notably, the etherification and rehydration product selectivity followed a fairly linear trend for HPW_4_Mo_10_O_*x*_, with reaction time (Table 1, entries 2 and 3). As Table 1, entry 3, show that dehydration and re-hydration of glucose to HMF and ring opening are a time-dependent reaction, ∼95% ring-opening product formed at 30 min. In our quest for a compatible catalyst, HPW_4_Mo_10_O_*x*_-Cys use for controlled rehydration of HMF and possessing the ability to selective dehydration of glucose to HMF and etherified product EMF. As shown in Table 1, entry 5, 50.12% HMF selectivity achieved, which is 5.6 times more than selectivity obtains from HPW_4_Mo_10_O_*x*_. As shown in Table 1, glucose conversion predominantly decided by the acidity of as-synthesized materials and times, whereas the distribution of dehydration and etherified products is evidently influenced by the acidity and morphological structure of the as-synthesis catalyst for glucose conversion. It is noteworthy that capping agent based HPA nanomaterials with lesser acid density (0.63 mmol g^−1^) exhibit higher HMF selectivity in the order to HPW_4_Mo_10_O_*x*_ (3D), higher acidic materials (0.84 mmol g^−1^). This result suggests HPW_4_Mo_10_O_*x*_ can favor the rehydration of furanics intermediate and the production of EL. Besides the acidity, the substrate, which is main dehydration intermediate (fructose), is another important factor affecting the conversion and selectivity of the desired product (Table 1, entries 6 and 7). We observed that HMF and the etherified product can be produced in high yields by HPW_4_Mo_10_O_*x*_ or HPW_4_Mo_10_O_*x*_-Cys catalyzed dehydration and etherification of fructose under microwave reactor using low boiling solvents (THF), reactive aqueous phase (water) and etherified solvent (ethanol). It suggests that the stronger acidity of HPW_4_Mo_10_O_*x*_ could cause the rehydration of EMF to EL (87.86%, Table 1, entry 6), whereas the weaker one of the HPW_4_Mo_10_O_*x*_-Cys is not beneficial to the rehydration of EMF or HMF, thus this is disfavour the selective production of EL (Table 1, entry 7). In summary, HPW_4_Mo_10_O_*x*_ is suitable for EL and EMF synthesis and HPW_4_Mo_10_O_*x*_-Cys, suitable for HMF synthesis due to the presence of strong and medium acid sites and 3-dimensional structure.

**Table 1:** One-pot catalytic dehydration and etherification of mono-sugar

S. no	Substrate	Catalyst	Time (min)	Conversion (%)	Yield^a^ (%)	Product distribution selectivity			
						HMF	EMF	EL	Others^b^
1	Glucose	A	10	84	56	22.45	42.68	12.82	6.10
2	Glucose	A	20	97	78	8.95	26.25	51.67	10.13
3	Glucose	A	30	∼100	73	0	2.40	84.95	11.60
4	Glucose	B	10	56	31	35.95	15.25	3.48	1.32
5	Glucose	B	20	92	76	50.12	26.76	7.10	7.20
6	Fructose	A	20	∼100	84	0	1.2	87.86	10.83
7	Fructose	B	20	∼98	89	54.65	28.80	7.40	7.15

a Isolated mol% yield.

b Other = LA, formylated HMF, FA. AHPW_4_Mo_10_O_*x*_, B= HPW_4_Mo_10_O_*x*_-Cys, other reaction condition: catalyst = 20 mg, temperature = 170 °C, solvent (6 : 2 : 2) THF : H_2_O : EtOH, substrate = 100 mg.

### 3.4 Effect of catalyst loading

**Fig. 9:**
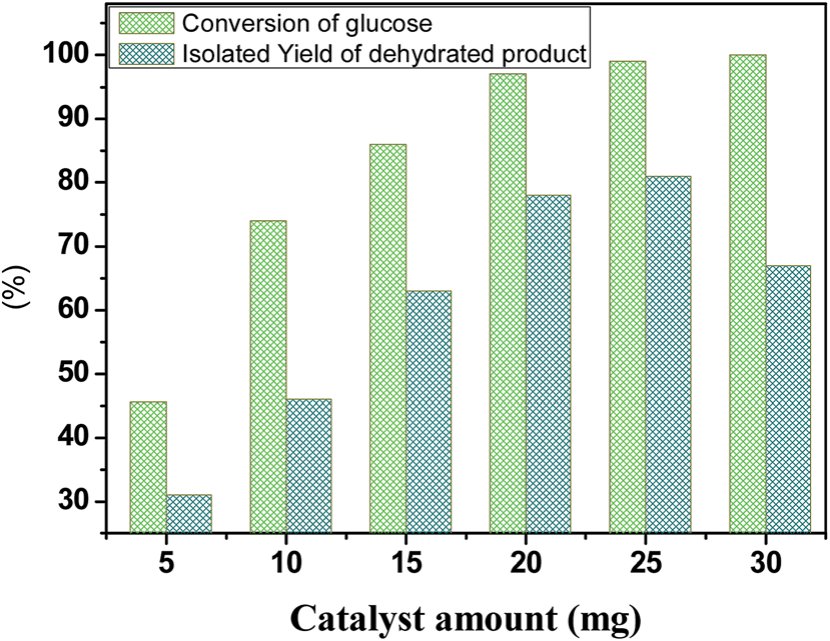
Dehydration and etherification of glucose at different catalyst loading, other reaction condition: glucose 100 mg, temperature 170 °C, time 20 min.

To further elucidate the catalyst amount of HPW_4_Mo_10_O_*x*_, a conversion of glucose monitored with different catalyst-dose, result shown in Fig. 9. Compared with 5 mg catalyst loading, 45.6% glucose conversion achieved with 31 mol% of isolated yield, moreover, when the catalyst-loading increased 10 mg, 74.01% conversion with 46 mol% of isolated yield occurred, we observed an even higher conversion of glucose (86%) and 97%, when catalyst-amount increased from 15 and 20 mg respectively with 78 and 67 mol% of isolated yield at 170 °C for 20 min, under microwave-irradiation. The increase in the glucose conversion and isolated yield with increasing catalyst loading could be attributed to an increase in the availability and number of catalytically active sites during dehydration and etherification of glucose. However, a further increment of catalyst loading 25 mg, no significant increment of yield (78 to 81%) achieved under same reaction condition. Further increment of 30 mg, decreasing trend of isolated product observed, indicating other side products formed like humins under a high acid concentration.

### 3.5 Comparative studies of alcohols for glucose conversion and respective etherification

**Fig. 10:**
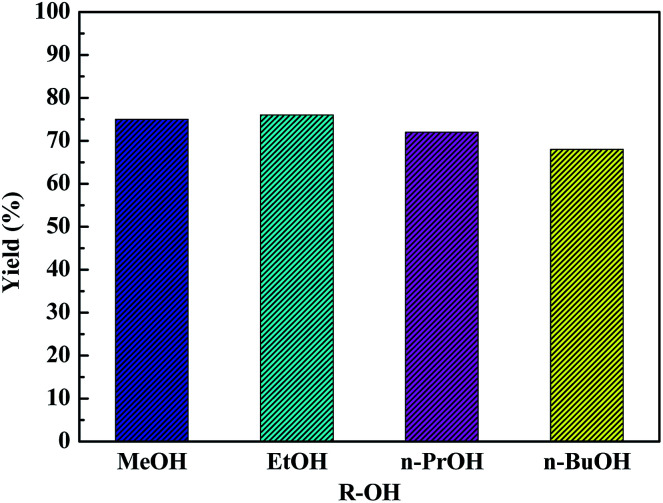
The catalytic effectiveness of HPW_4_Mo_10_O_*x*_-Cys for the selective dehydration of glucose and further etherification with various aliphatic alcohols. Reaction conditions: glucose = 100 mg, catalyst 20 mg, time = 20 min.

The versatility of as-synthesized HPW_4_Mo_10_O_*x*_-Cys catalyst towards dehydration and etherification reactions was further explored using methanol, *n*-propanol, isopropanol, *n*-butanol, and 2-butanol (Fig. 10). Ethanol and methanol showed a similar conversion and isolated yield, whereas propanol, isopropanol, *n*-butanol, and 2-butanol were less reactive compared to ethanol. Since, utilization of higher alcohol and synthesis of respective AMF and AL from glucose is an exciting development in the production of higher chain furanics and its derivative, which would have higher energy density and find wider application as solvent, plasticizing agent and other bio-based chemicals. The results show in Fig. 10, tolerance and effectiveness of HPW_4_Mo_10_O_*x*_-Cys catalyst with methanol (75%, 96%), *n*-propanol (72%, 89%) and *n*-butanol with (68, 72%) mol% yield and conversion of glucose.

### 3.6 Effect of substrate

The effectiveness of the catalyst was further evaluated by the exploring some more complex sugar to intermediate platform chemicals at optimum condition. A reaction under the condition of 100 mg of starting substrate, 20 mg of catalyst loaded in microwave tube, closed and set run for 20 min at 170 °C, achieved quantitative conversion of fructose, sucrose, and HMF were more than 99% with 84, 81 and 89% of desired product yield (Table 2, entries 1, 3, and 5). Other complex biomass (crystalline cellulose) was tested for dehydration and etherification reaction, we are failed to achieve desired product at optimised reaction condition. Maybe due non-solubility of cellulose in water or THF. Therefore, we were added 10% ionic liquid (1-allyl-3-methylimidazolium chloride) for the dissolution of cellulose at optimum conditions and 67% conversion and 42% of the desired yield achieved (Table 2, entry 7). We have also tested furfuryl alcohol as starting substrate for etherification reaction, and 92% conversion with 87% isolated yield was achieved. Although fructose is the preferred raw materials for dehydration and etherification, its occurrence in nature is limited. This drives our more intrest to use a more abundant carbohydrate fraction, glucose, as the starting materials for further reaction.

**Table 2:** Substrate scope for dehydration and etherification

S. no	Substrate	Conversion (%)	Isolated yield (%)
1	Fructose	>99.9	84
2	Glucose	92	76
3	Sucrose	∼99	81
4	Furfural alcohol	92	87
5	HMF	>99.9	89
6	LA^a^	>99.9	93
7	Cellulose^b^	67	42

a Esterification.

b 10 mol% ILs was used, other reaction parameter are same.

### 3.7 Reusability of catalyst

**Fig. 11:**
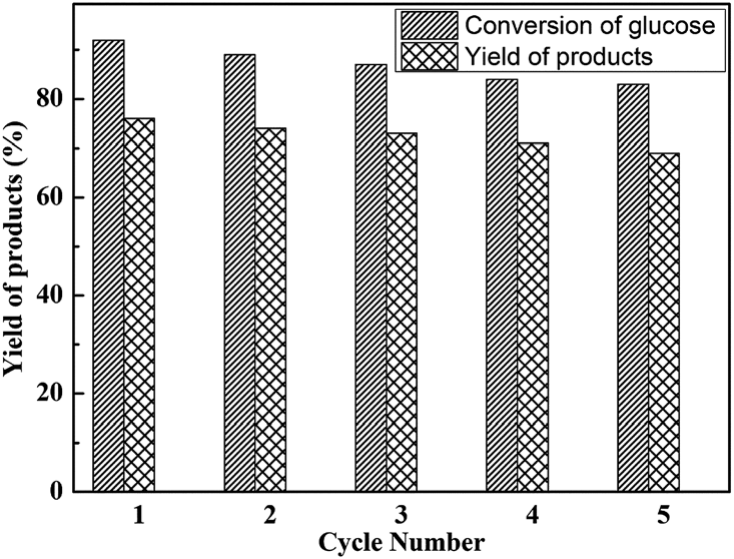
Reusability study of HPW_4_Mo_10_O_*x*_-Cys catalyst for glucose dehydration and yields of products. Other reaction condition: glucose = 100 mg, catalyst = 20 mg, at 170 °C for 20 min.

To further analysis the stability of the as-synthesized and heterogenized HPW_4_Mo_10_O_*x*_-Cys catalyst, with time on dehydration and etherification of glucose was successfully tested for five consecutive cycles. Recycling experiments show comparable activity of HPW_4_Mo_10_O_*x*_-Cys, in terms of glucose conversion and EMF yield, in the first cycle under optimal reaction conditions (Fig. 11). The reaction was carried out at 170 °C under the MW-irradiation for 20 minute duration with 20 mg of catalyst and 100 mg of glucose. After each cycle, the catalyst was filtered, washed and activated through calcinations before used for next cycle. The next cycle using the recovered catalyst was carried out under similar condition by adding fresh glucose and THF, ethanol, and water as solvent (6 : 2 : 2) ratio. As the end of the reaction, the product was analyzed by GC, HPLC, UV and NMR. The results as shown in Fig. 11 suggest that the activity of catalyst after five cycles, in terms of conversion and selectivity results demonstrated that the only 9% conversion and 7% yield of desired product decreased after five cycles.

### 3.8 UV-visible method

The UV-visible spectrum of pure HMF solution has a distinct peak at 286 and 228 nm in water with corresponding molar extinction coefficient (*ε*) value of 1.66 × 10^−4^ M^−1^ cm^−1^.

**Fig. 12:**
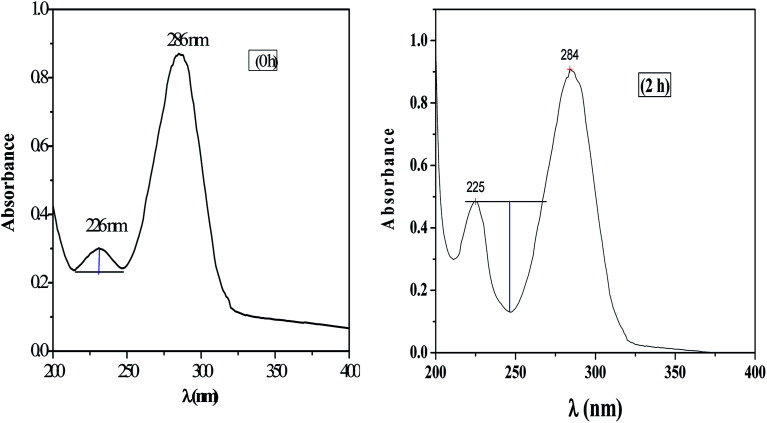
UV absorption spectra for the HMF at zero h and after etherification (2 h).

HMF has two functional group, carbonyl, and alcohol, and therefor in carbonyl group (n to π and π to π*) transition are possible and in alcohol (n to σ*) transition possible. UV absorption spectra for HMF show two transition to π* and π to π*. Characteristic absorption peaks of HMF (286 nm) after etherification as shown in Fig. 12. The spectra of pure HMF, and after etherification were easily distinguished by UV-visible. Results indicate that the absorption due to π to π* transition decrease (286 nm) along with increasing n to π transition intensity (225 nm) (Fig. 12, 2 h). As the reaction time increases beyond this initial period, the UV spectra begin to show an increase in absorbance at wavelengths lower than 230 nm, along with a decrease in absorbance observed for wavelengths above 284 nm and therefore no appearance of alcohol peak. Fig. 12 shows a spectrum of HMF at zero h and 2 h, in view of the spectra where increase in absorbance can be seen as deepness of the valley increased.

### 3.9 Techno-economic analysis

The biorefinery based chemical is a green concept advancing chemistry and chemical engineering to synthesize and use biofuels in place of fossil fuels (oil and gas). The major focus of this research work is to developed cost-effective catalytic route for the synthesis of renewable sources of energy that can be competitive with fossil fuels. The US Department of Energy selected top-10 biomass-based chemicals on the basis of the well-stabilized reaction process, economics, industrial viability, size of markets and ability the compound to serve as a platform chemicals for the transformation of other valuables chemicals and fuels.^1^ The as-synthesized materials show the promising result in these regards, as shown in Table 2, that anchored HPA has a better selectivity and efficiency and relatively low humins formation. Accordingly, we designed a process and conducted a techno-economic study to determine the feasibility of using the HPW_4_Mo_10_O_*x*_ and HPW_4_Mo_10_O_*x*_-Cys as catalysts, in comparison to mineral acid, and nano-material as catalyst for carbohydrate dehydration, etherification, and rehydration. In comparison to biomass transformation published literature our catalytic results reveal that as-synthesized materials have better acidity controlled and better efficiency to transform the carbohydrate to valuable chemicals and fuel at economical cost and sustainable manner and attuned with the fossil-based market. Our materials design for catalytic reaction and analysis studies conclude that: (i) catalytic route design for the synthesis of HMF, EMF and alkyl levulinate from carbohydrate can be easily scalable up to pilot-plant level. (ii) Utilization of MW energy can reduce the reaction time and energy per capita cost. (iii) Utilization of glucose and sucrose as starting substrate can also, decrease the starting raw materials cost. (iv) True heterogeneous nature and better recovery of the catalyst after reaction can provide the relatively lower cost of materials and total production cost. The heating value of various alcohol and furanics mentioned in Table 3, indicate that furanics have potential to replace the fossil fuel in the future.^55^

**Table 3:** Comparative energy density of gasoline, alcohols, and biomass-derived furanics^55^

Compound	Lower heating value (MJ L^−1^)	Boiling point (°C)	RON
Gasoline	30–33	27–225	88–98
Ethanol	21.4	78	109
*n*-Propanol	24.7	97.2	104
Iso-propanol	24.1	82.3	106
*n*-Butanol	26.9	117.7	98
Iso-butanol	26.6	107.9	105
*n*-Pentanol	28.5	137.8	78
MTHF	28.2	78	86
DMF	30.1	94	119
MF	27.6	64.7	103
EL	24.8	206	110
BL	27.1	237.5	98
MP	25.9	126	105
GVL	26.2	218–220	100

a MTHF = 2-methyltetrahydrofuran, DMF = 2,5-dimethylfuran, MF = 2-methylfuran, EL = ethyl levulinate, BL = butyl levulinate, MP = methyl pentanoate, GVL = γ-valerolactone.

## 4. Conclusion

Highly stable, 3D hexagonal hollow core–shell structure of HPA has been synthesized. The use of capping agent, changes in the morphology is obtained. Structural characterization and acidity measurement show extremely good control of acidity and morphology achieved by using cysteine as a capping agent. The dehydration and etherification reactions show different selectivity of the product by using HPW_4_Mo_10_O_*x*_ and HPW_4_Mo_10_O_*x*_-Cys catalyst. The NMR and GC results studies suggest that the HPW_4_Mo_10_O_*x*_-Cys material is capable for selective dehydration mono-sugars to HMF and HPW_4_Mo_10_O_*x*_ materials show high degree of Brønsted and Lewis acidity to formHMF and LA. It can further enhance the selectivity of rehydration product (AL) at the cost of HMF/AL at higher reaction time. Therefore as-synthesized materials were effective catalysts for the conversion of monosugar or furanics intermediate into biofuels additives because of their Brønsted acidity and well-defined structure, high proton mobility and ability to accepter–donor of electrons. Furthermore, high catalytic activity, good thermal stability and easy separations of as-synthesis HPW_4_Mo_10_O_*x*_-Cys act as a true promising bifunctional heterogeneous solid acid catalyst.

## Conflicts of interest

There are no conflicts to declare.

## Supplementary Material

RA-010-C9RA03300A-s001
