# Supplementary material for: Topotactic transformation of homogeneous phosphotungastomolybdic acid materials to heterogeneous solid acid catalyst for carbohydrate conversion to alkyl methylfurfural and alkyl levulinate
Source: RSC Adv. 2020 Jan 2;10(2):705–18. doi: 10.1039/c9ra03300a (PMC9048189; doi:10.1039/c9ra03300a)
Supplement: RA-010-C9RA03300A-s001 [file RA-010-C9RA03300A-s001.pdf]

Electronic Supplementary Material (ESI) for RSC Advances.
This journal is © The Royal Society of Chemistry 2019

Topotactic transformation of homogeneous phosphotungastomolybdic acid materials to heterogeneous solid acid catalyst for carbohydrate conversion to alkyl levulinate

Dinesh Gupta*[a], Chandrakant Mukesh[a], Kamal K. Pant*[a],
Chemical Engineering, Indian Institute of Technology Delhi, New Delhi 110 016, India
Corresponding Author: email: dineshguptagkp@gmail.com kkpant@chemical.iitd.ac.in

Both ^{1}H and ^{13}C NMR spectra of the product exposed the formation of EMF and EL as the sole products, and their observed characteristic signals in the NMR spectra are as follows; EMF (^{1}H-NMR-$CDCl_3$): δ 9.614 (s, 1H), 7.19 (d, J= 3.99 Hz, 1H), 6.50 (d, J= 3.99 Hz, 1H), 4.50 (s, 2H), 3.57 (q, 2H), 1.22 ppm (t, 3H); ^{13}C-NMR ($CDCl_3$): δ 15.03, 64.72,66.61,111.00,122.022,152.50,158.74,177.75 ppm. EL (^{1}H-NMR-$CDCl_3$): δ 4.10 (q, 2H), 2.73 (t, 2H), 2.53 (t, 2H), 2.16 (s, 3H),1.21 ppm (t,3H); ^{13}C-NMR ($CDCl_3$): δ 14.12,27.97, 29.55,37.90,60.61,173.0,207.4 ppm.

The solid mixture are dissolve in $CDCl_3$ along with international standard mesitylene, run the NMR spectra: EMF (^{1}H-NMR-$CDCl_3$): δ 9.584 (s, 1H), 7.19 (d, J= 3.99 Hz, 1H), 6.50 (d, J= 3.99 Hz, 1H), 4.50 (s, 2H), 3.57 (q, 2H), 1.22 ppm (t, 3H); ^{13}C-NMR ($CDCl_3$): δ 15.03, 64.72,66.61,111.00,122.022,152.50,158.74,177.75 ppm. EL (^{1}H-NMR-$CDCl_3$): δ 4.10 (q, 2H), 2.73 (t, 2H), 2.53 (t, 2H), 2.16 (s, 3H),1.21 ppm (t,3H); ^{13}C-NMR ($CDCl_3$): δ 14.12,27.97, 29.55,37.90,60.61,173.0,207.4 ppm. HMF (^{1}H-NMR-$CDCl_3$): δ 9.50 (s, 1H),7.17 (d, J= 4.39 Hz, 1H), 6.50 (d,J=4.39, 1H), 4.66 (d,3.39 Hz, 1H)

HPLC analysis of remaing sugars and dehydrated and rehydrated product:

Glucose, fructose, HMF, EMF, and 5-HMF were analyzed using high-performance liquid chromatography (HPLC). HPLC analysis was performed using Agilent technology 1200 infinity equipped with Bio-Rad Aminex HPx-87H 300 7.8 mm columns, UV-detector for sugar dehydration product and RI detector for sugar dehydration products. The eluent was 5 mM sulphuric acid with a flow rate of 0.6 mL per minute and the injection volume was 20 μL. Low boiling product were analyzed in gas chromatograph (Nucon 5700) equipped with FID and TCD detector. The liquid product were analyzed in GC-TCD after separation of catalyst,after dilution of 20 μL product solution in 1 mL methanol. The liquid products were also analyzed in GC–MS equipped with DB-5 column.The identification of products in the sample was performed using a pure form of the products to determine retention time and calibration curve formation. The 5-HMF, AML and AL were calculated as given below in equation.

$$Yield\ of\ product\ (Mol\ \%) = \frac{Mol\ of\ product}{Mol\ of\ starting\ substrate} * 100$$

$$Conversion\ (\%) = \frac{Glucose\ in\ \text{-}\ glucose\ out}{Total\ glucose\ in} * 100$$

$$Selectivity\ (\%) = \frac{Mol\ \%\ yield\ of\ desired\ product}{total\ yield\ of\ all\ desired\ product} * 100$$

NMR Spectra of as-synthesized product

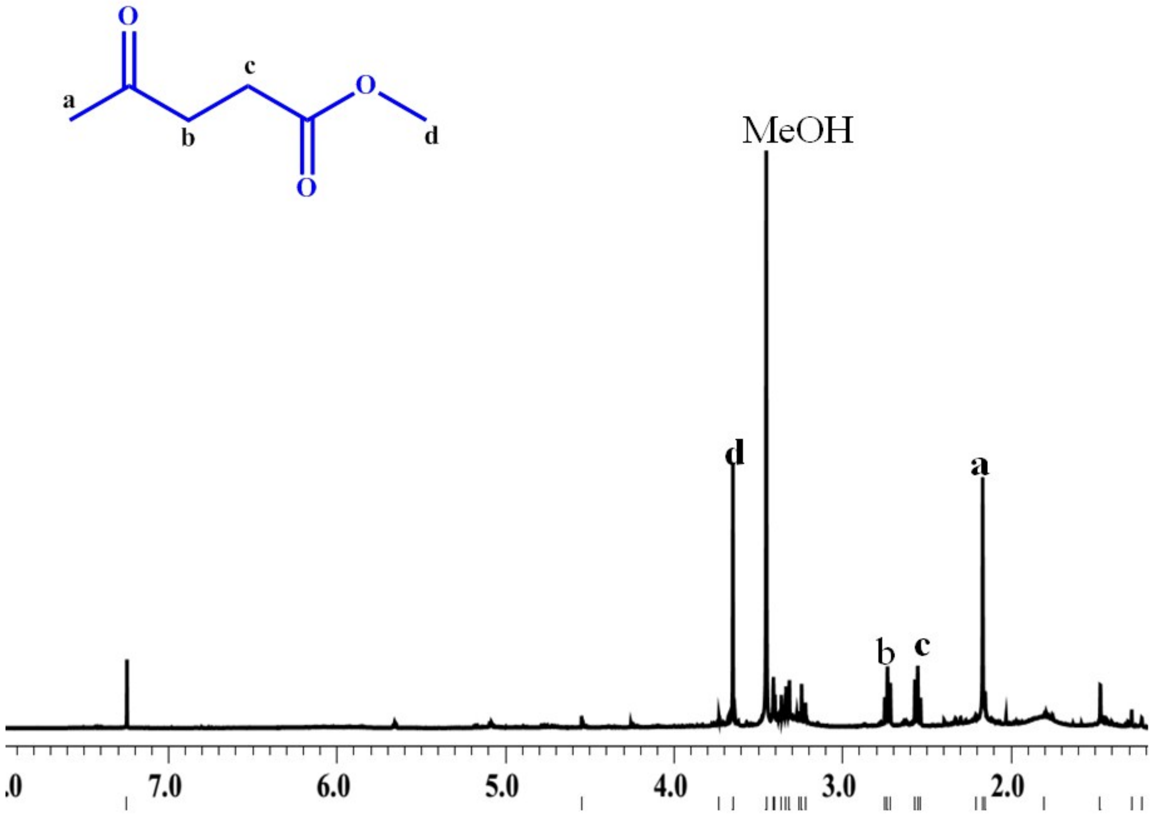

Fig. S1: ^{1}H-NMR of ML

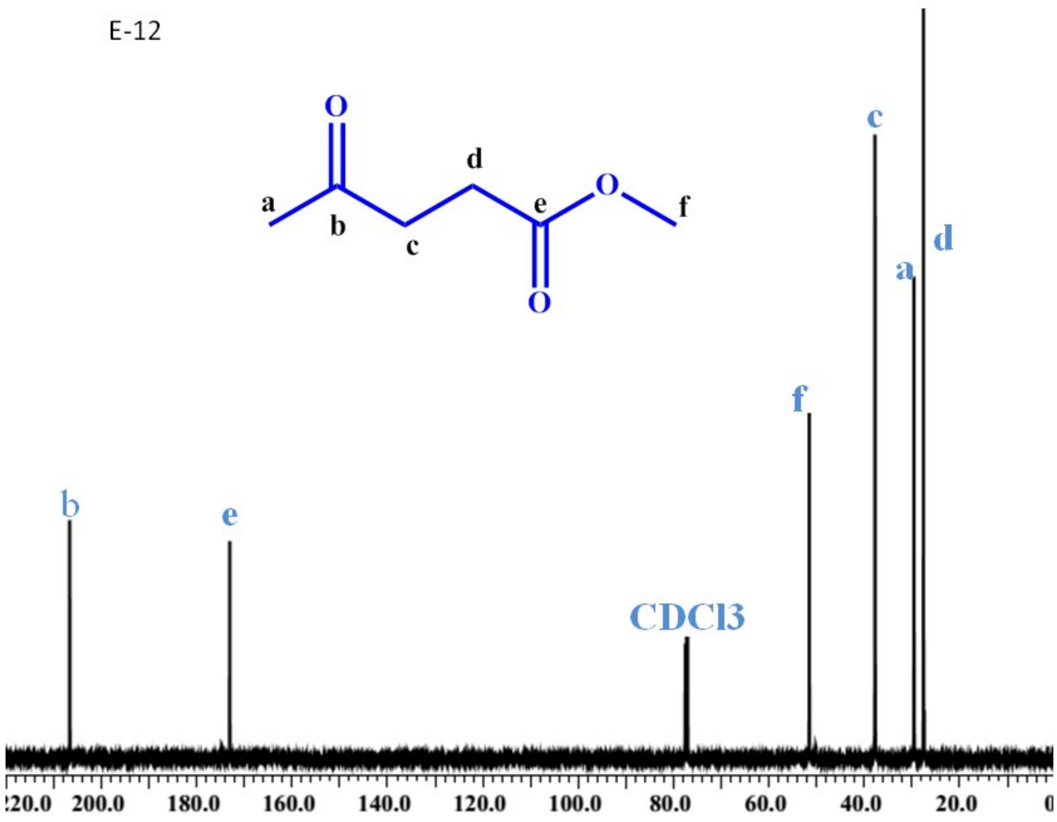

Fig. S2: ^{13}C-NMR of ML

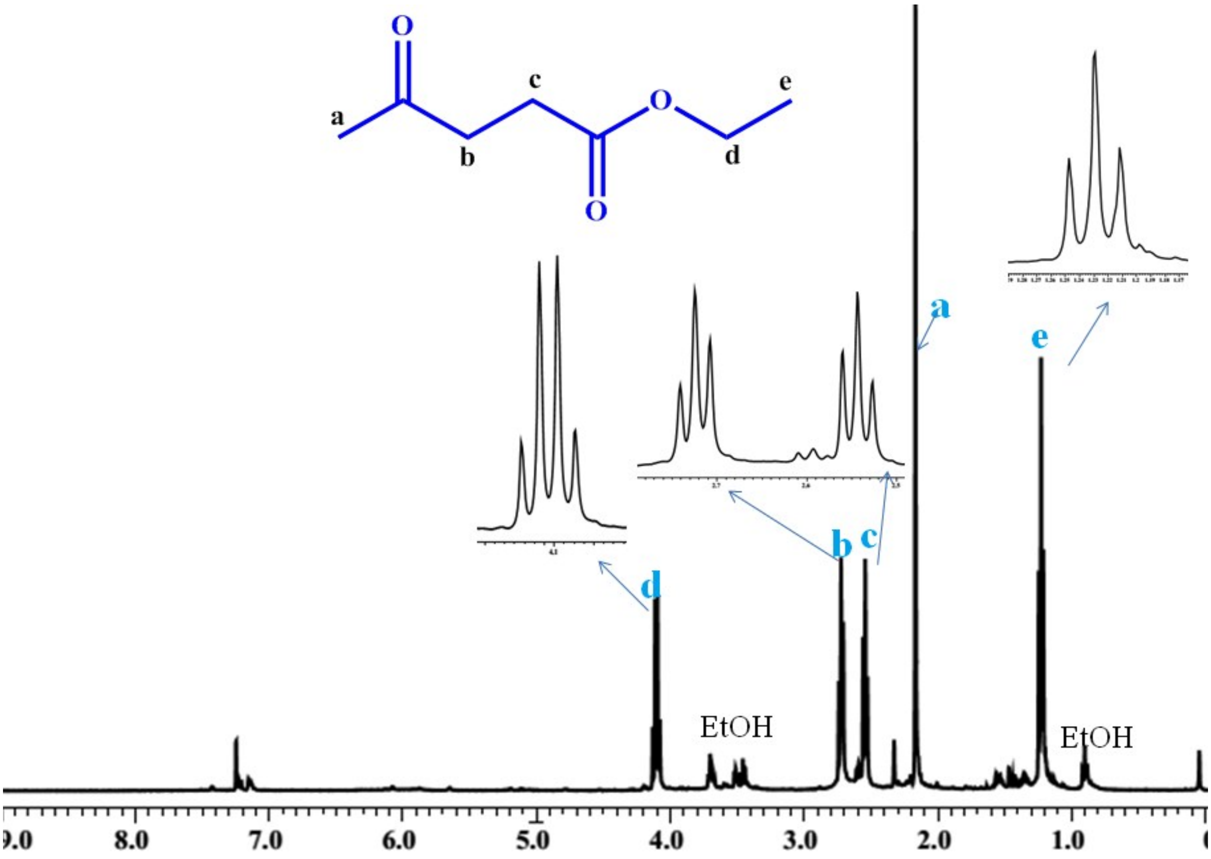

Fig. S3: ^{1}H-NMR of EL

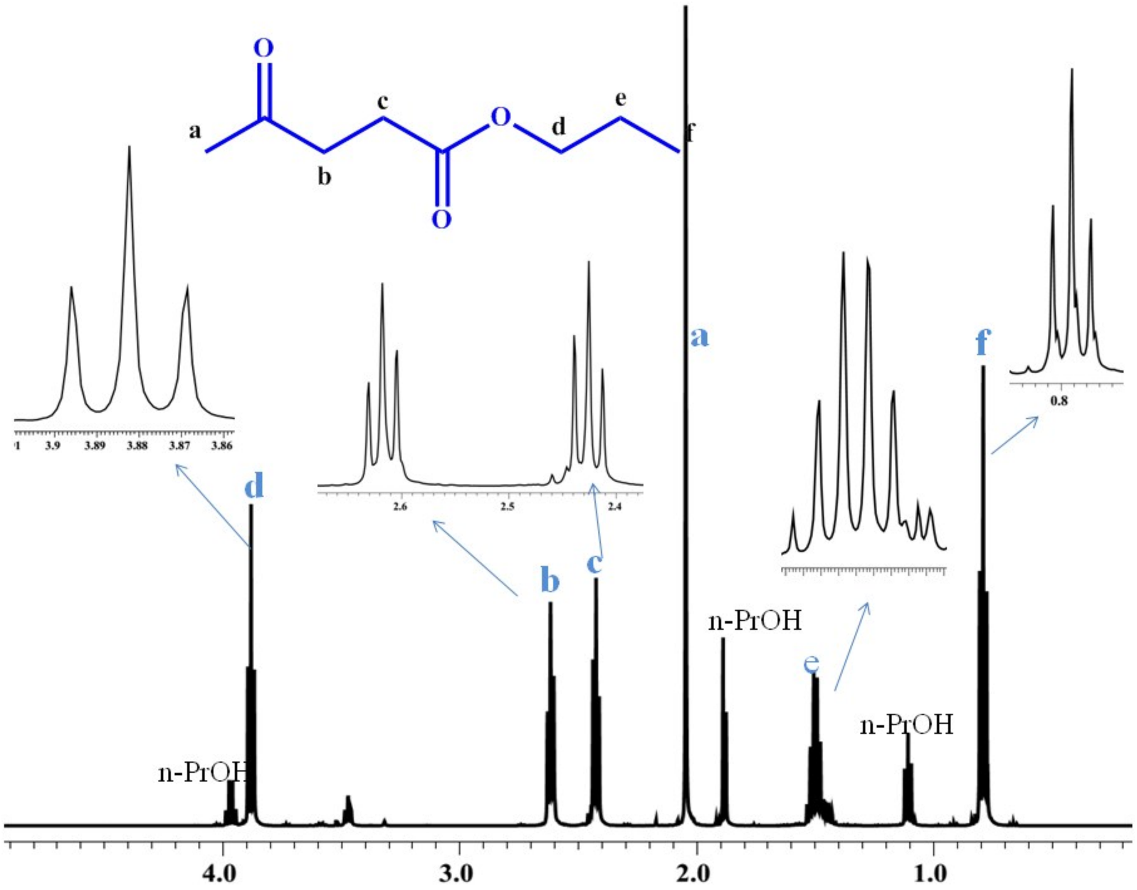

Fig. S4: ^{1}H-NMR of PL

-

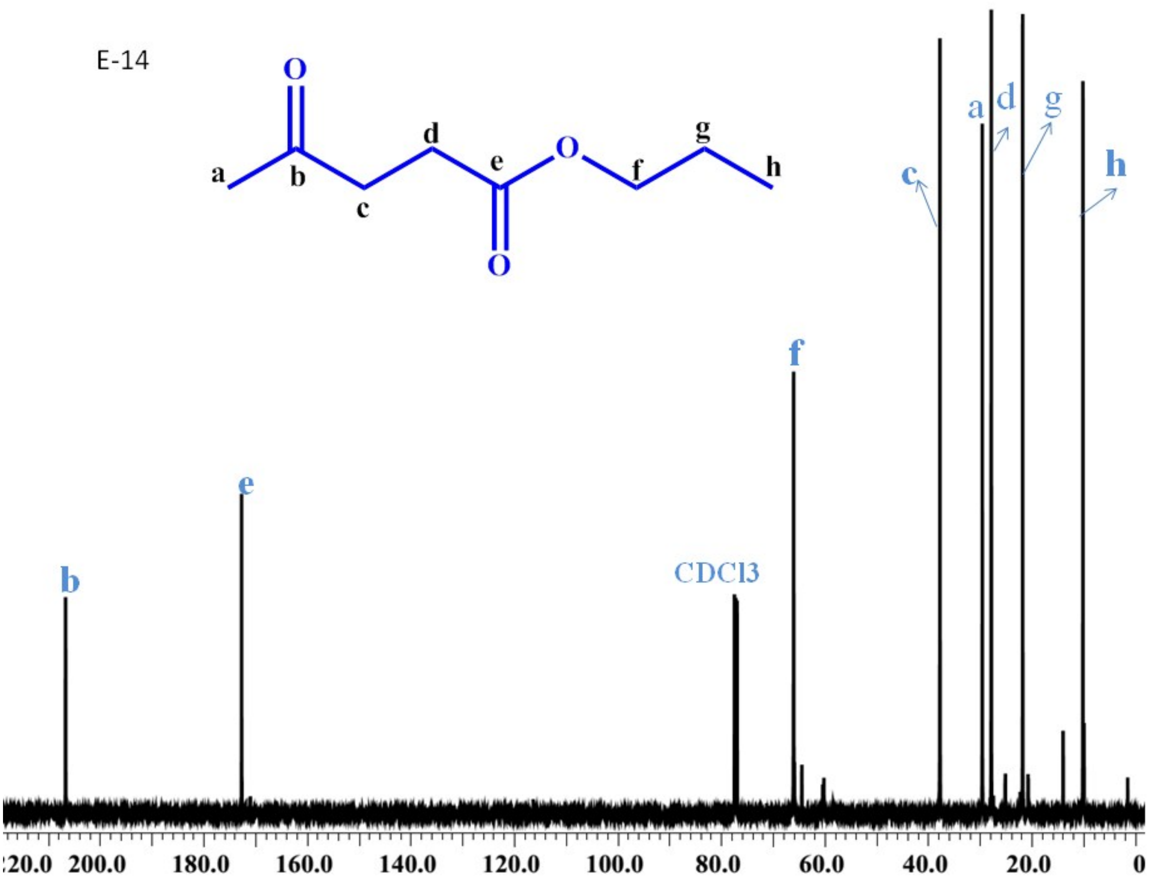

Fig. S5: ^{13}C-NMR of PL

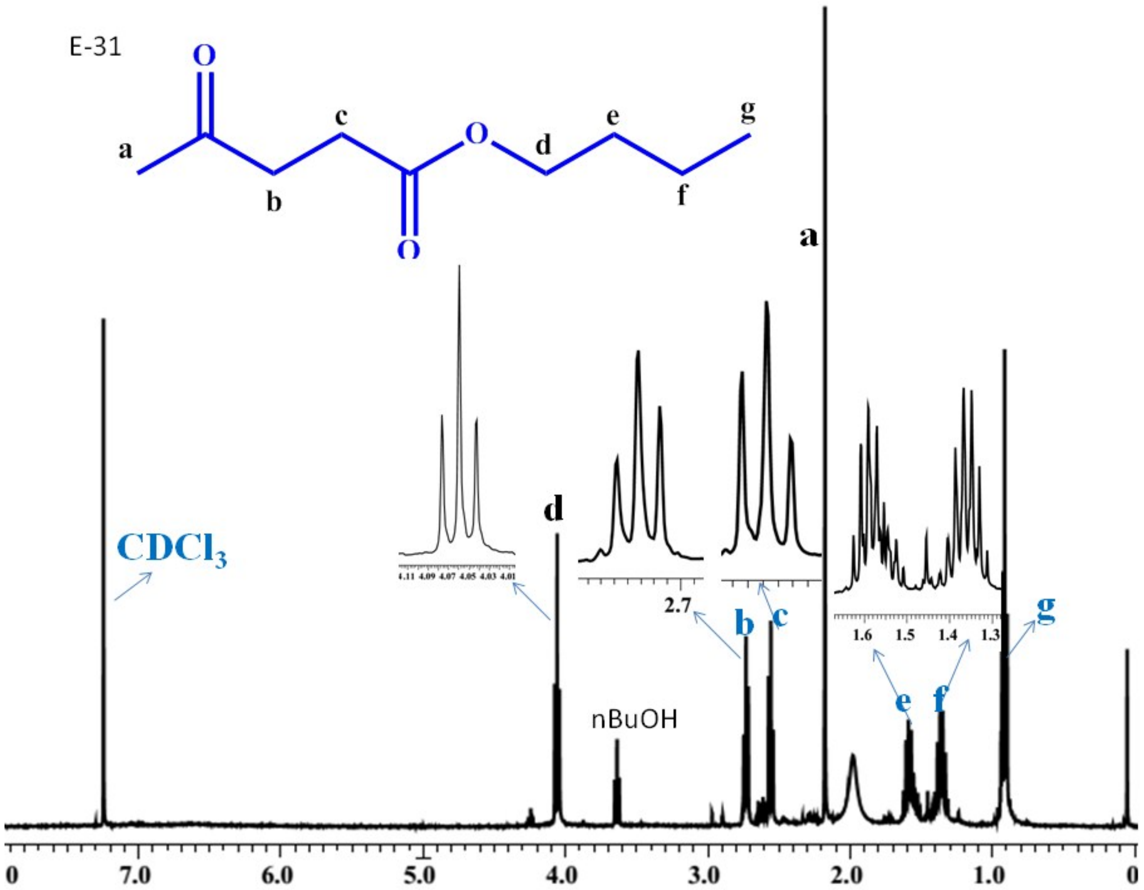

Fig. 6: ^{1}H-NMR of BL

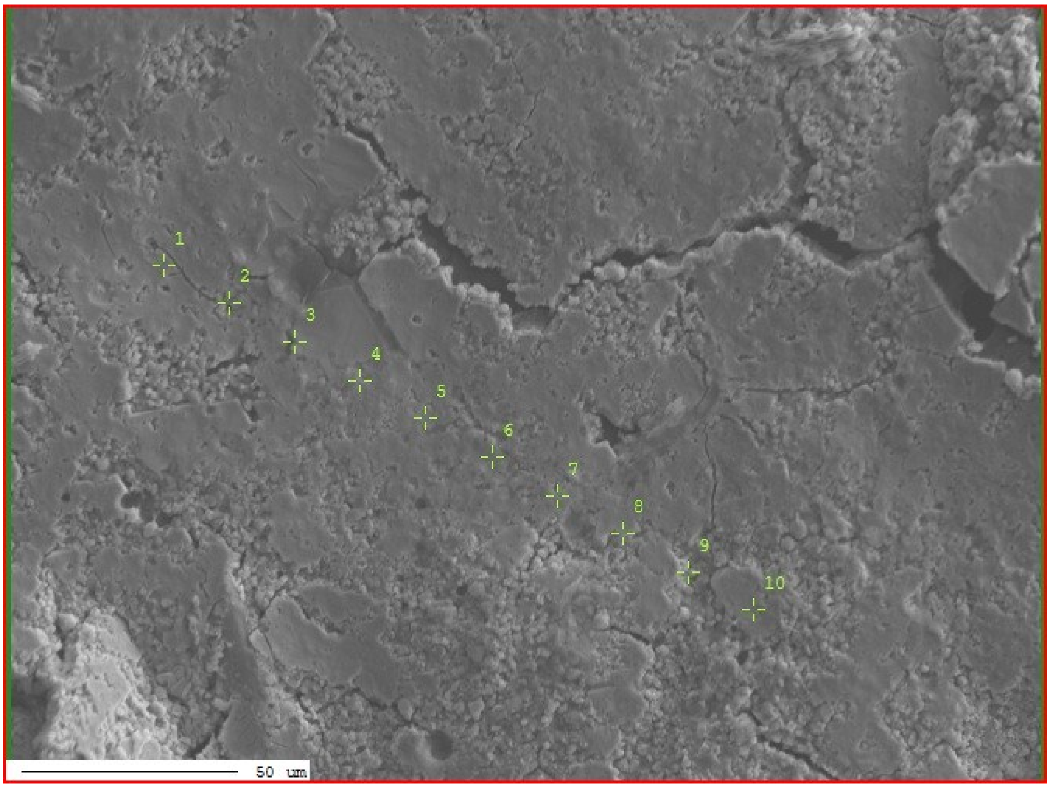

Fig.S7: Secondary electron image of as synthesized $HPW_4Mo_{10}O_x$ -Cys. Analysed positions for EPMA are indicated in the picture

Table S1 : 10 point elemental mapping of as-synthesized $HPW_4Mo_{10}O_x$ -Cys materials

Element → Point ↓	P	O	N	Mo	W
	Weight %				
1	1.52	14.85	0.32	32.15	23.79
2	0.53	8.96	0.91	9.47	7.303
3	1.79	14.40	0.00	33.25	22.04
4	1.51	20.45	1.49	31.80	24.34
5	1.40	10.65	0.00	27.70	19.72
6	1.58	14.87	0.00	31.80	23.45
7	1.54	18.71	0.00	32.87	25.29
8	1.72	13.16	0.02	34.27	24.29
9	0.49	4.04	0.22	8.84	6.66
10	1.65	22.33	0.91	32.15	28.02

Table S2 : 10 point elemental mapping of as-synthesized $HPW_4Mo_{10}O_x$ materials

Element → Point ↓	P	O	Mo	W
	Weight %			
1	1.45	24.52	28.31	35.05
2	1.45	24.27	28.75	34.47
3	1.35	24.74	26.43	34.41

4	1.29	17.01	23.43	37.20
5	1.29	17.48	24.71	31.66
6	1.13	10.76	22.72	32.97
7	1.39	25.66	27.29	33.55
8	1.45	25.54	28.96	33.67
9	1.38	22.33	28.39	33.99
10	1.47	25.68	29.69	28.02

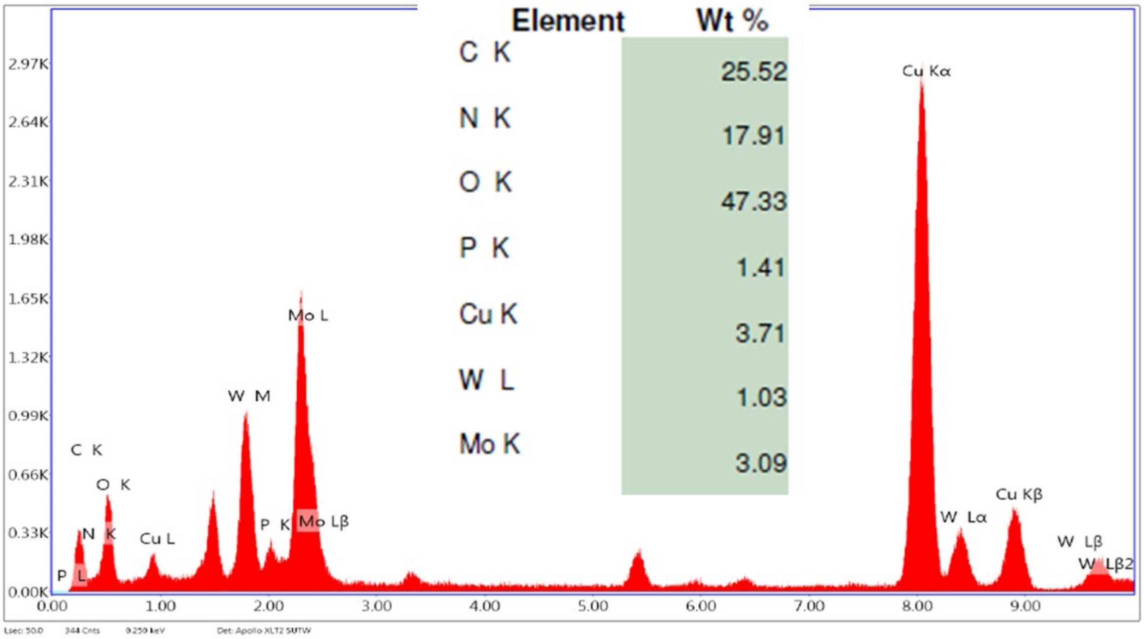

Fig. S8: Elemental analysis by TEM-EDX of HPMoWOx

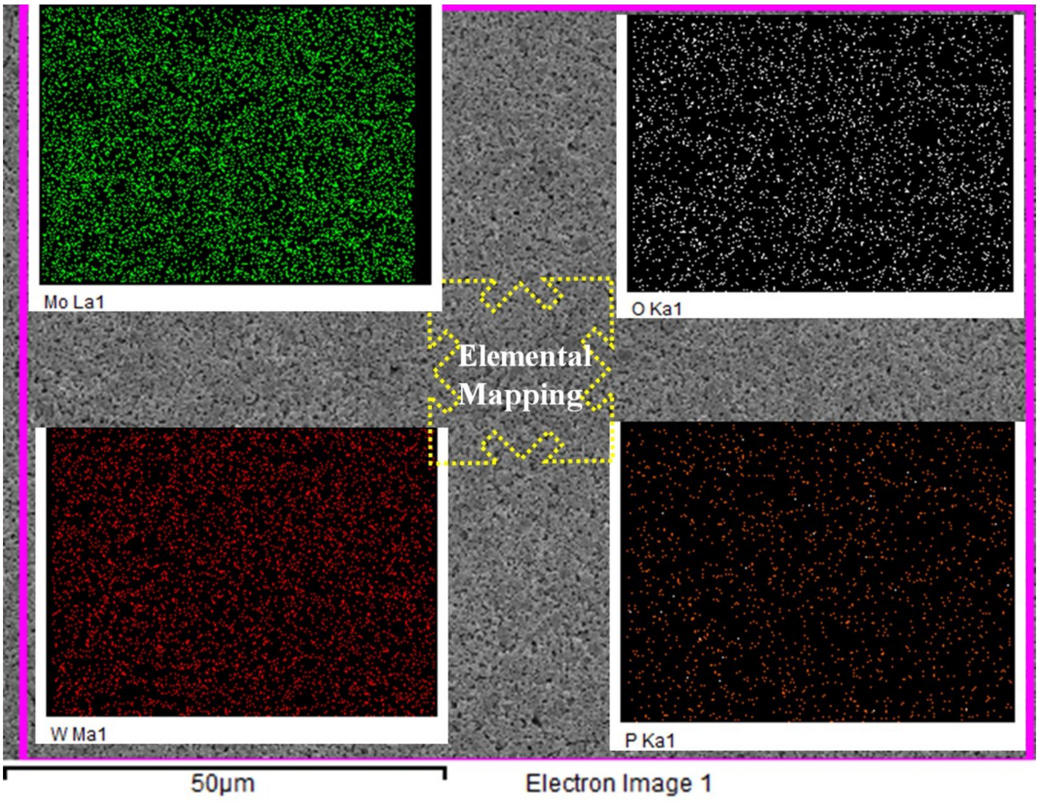

Fig. S9: FESEM-Elemental Mapping of as-synthesis materials

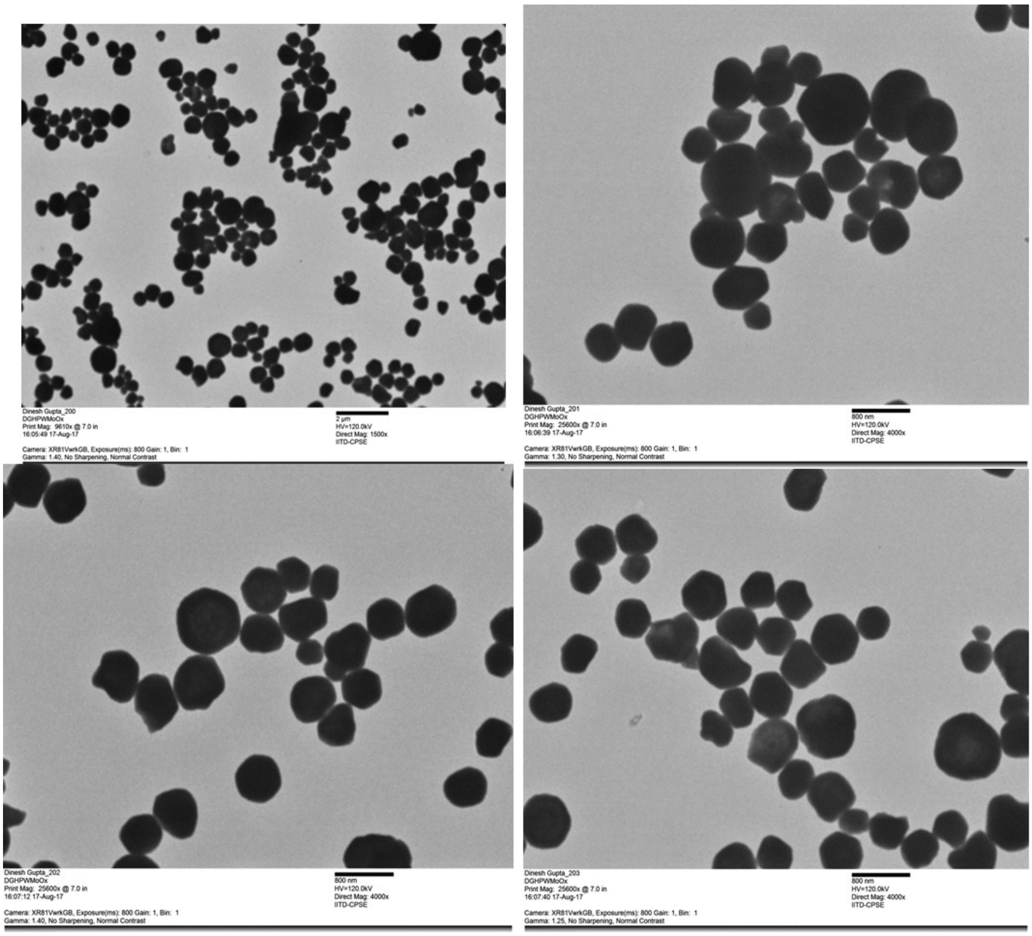

Fig. S10: Some more TEM images of as-synthesis materials

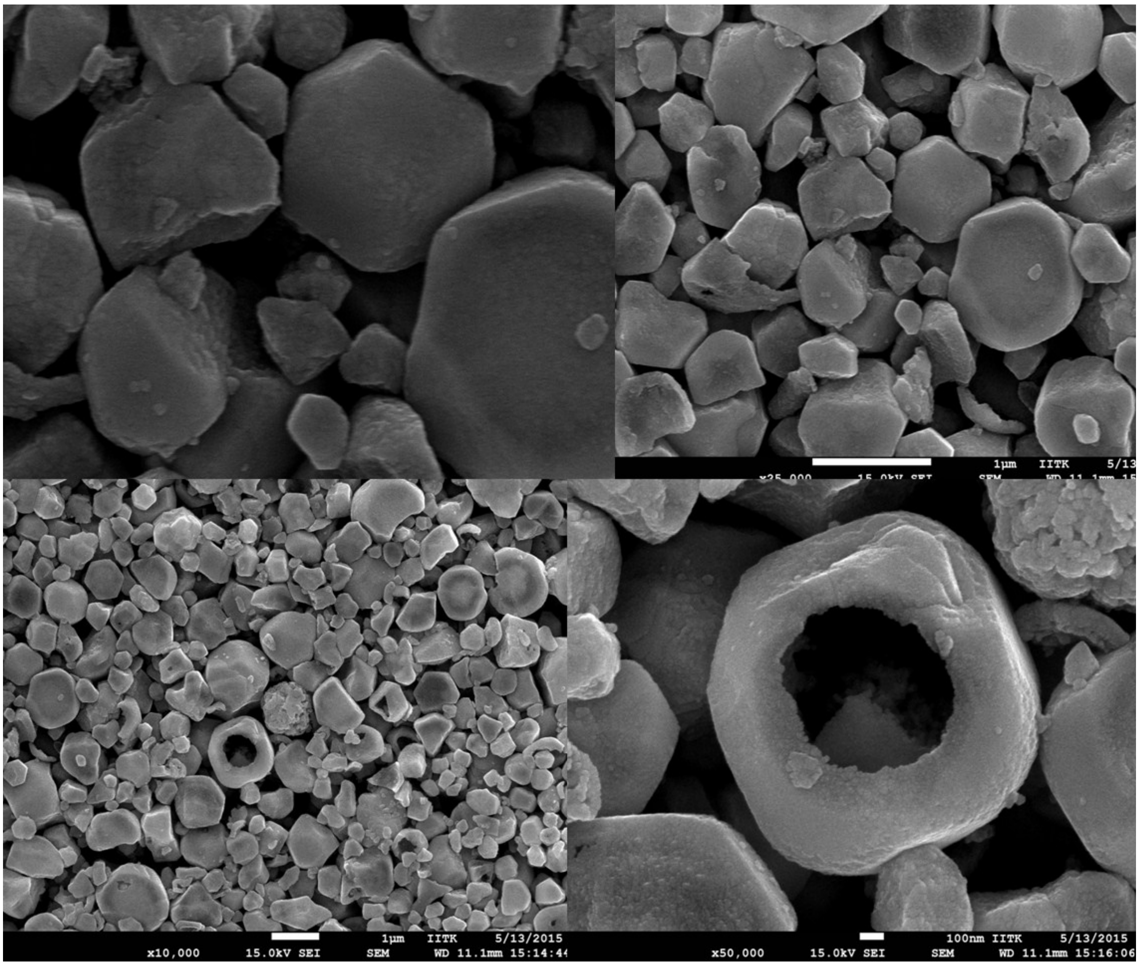

Fig. S11: Some more FESEM images of as-synthesis materials

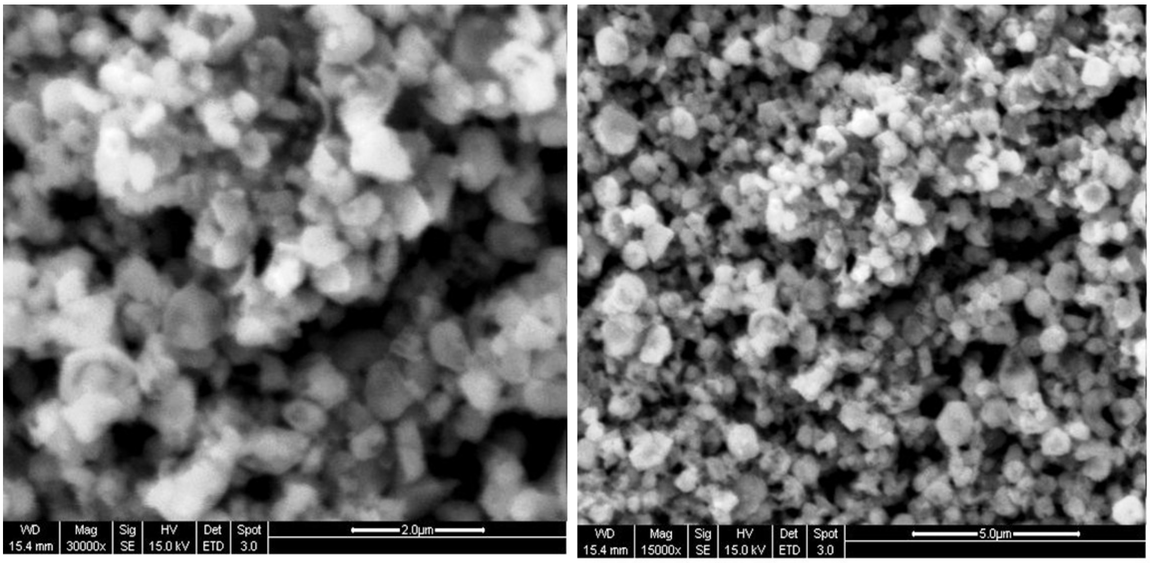

Fig. S12: Some more SEM images of as-synthesis materials

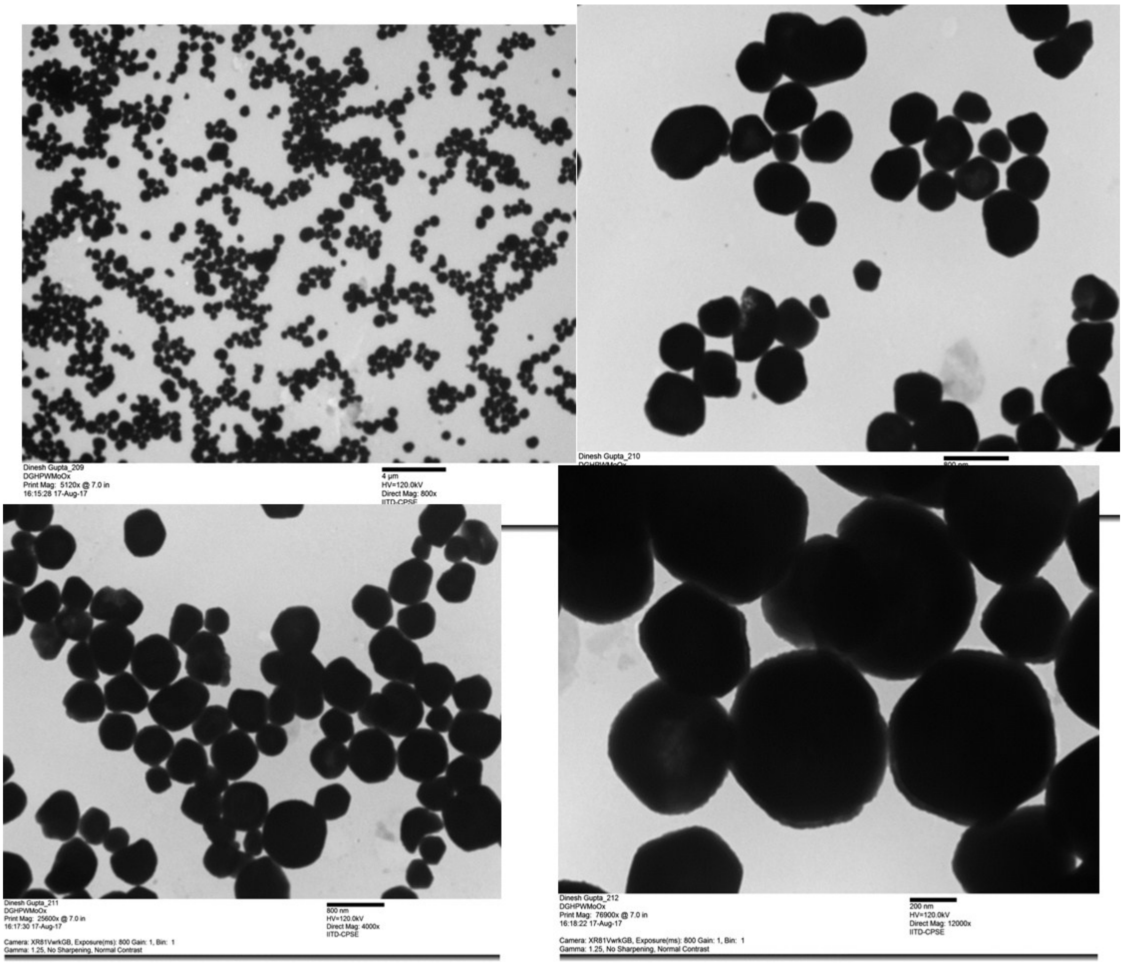

Fig. S13: Some more TEM and HRTEM images of as-synthesis materials.
